# Associations between dietary factors and obesity-related biomarkers in healthy children and adolescents - a systematic review 

**DOI:** 10.1186/s12937-017-0300-3

**Published:** 2017-12-28

**Authors:** Jennifer Hilger-Kolb, Catherin Bosle, Irina Motoc, Kristina Hoffmann

**Affiliations:** Mannheim Institute of Public Health, Social and Preventive Medicine, Medical Faculty Mannheim, Heidelberg University, Ludolf-Krehl-Str. 7-11, D-68167 Mannheim, Germany

**Keywords:** Dietary intake, Macronutrients, Biomarkers, Obesity, Children and adolescents

## Abstract

**Background:**

The obesity prevalence in children and adolescents has increased worldwide during the past 30 years. Although diet has been identified as one risk factor for developing obesity in this age group, the role of specific dietary factors is still unclear. One way to gain insight into the role of these factors might be to detect biomarkers that reflect metabolic health and to identify the associations between dietary factors and these biomarkers. This would enable nutrition-related metabolic changes to be detected early in life, which might be a promising strategy to prevent childhood obesity. However, existing literature offers only inconclusive evidence for diet and some of these obesity-related biomarkers (e.g., blood lipids). We thus conducted a systematic literature review to further examine eligible studies that investigate associations between dietary factors and 12 obesity-related biomarkers in healthy children and adolescents aged 3-18 years.

**Methods:**

We searched the scientific databases PubMed/Medline and Web of Science Core Collection for potentially eligible articles. Our final literature search resulted in 2727 hits. After the selection process, we included 81 articles that reported on 1111 single observations on dietary factors and any of the obesity-related biomarkers.

**Results:**

Around 81% of the total observations showed nonsignificant results. For many biomarkers we did not find enough observations to draw clear conclusions on possible associations between a dietary factor and the respective biomarker. In cases where we identified enough observations, the results were contradictory. Since these nonsignificant and inconclusive findings may impede the development of effective strategies against childhood obesity, this article takes a closer look at possible reasons for such findings. In addition, it provides action points for future research efforts.

**Conclusions:**

In conclusion, current evidence on associations between dietary factors and obesity-related biomarkers is inconclusive. We thus provided an overview on which specific limitations may impede current research. Such knowledge is necessary to enable future research efforts to better elucidate the role of diet in the early stages of obesity development.

**Electronic supplementary material:**

The online version of this article (10.1186/s12937-017-0300-3) contains supplementary material, which is available to authorized users.

## Background

The obesity prevalence in children and adolescents has increased worldwide during the past 30 years [1, 2]. According to the BMI cut-off-points of the International Obesity Task Force worldwide about 10% (155 million) of children and adolescents aged 5-17 years old were estimated to be overweight in 2004 [3, 4]. Moreover, among those 2-3% (30-45 million) were estimated to be obese [4, 5]. In the pediatric age group, obesity is associated with significant health consequences, such as hypertension, dyslipidemia, insulin resistance, and diabetes [1, 2]. Furthermore, it is an important risk factor for adult morbidity and mortality [6].

Diet is considered to play a key role in obesity development [7], and researchers worldwide have undertaken efforts to clarify the role of specific dietary factors in the complex etiology of obesity [8]. However, it is still unclear which nutrients and foods contribute to the development of obesity in children and adolescents [9]. This might be one reason for the limited success of existing obesity prevention strategies, as these strategies mainly focus on dietary behavior either alone or in combination with physical activity [10, 11].

One strategy to obtain new insights into the complex role diet is playing in obesity development may be the identification of biological markers that reflect metabolic health. Determining associations between dietary factors and such biomarkers could be helpful in detecting nutrition-related metabolic changes early in life, thus providing new pathways in the fight against obesity.

Former literature reviews already focused on some of these biomarkers. For example, a systematic review investigated associations between protein intake and three biomarkers of cardiovascular health: blood pressure, insulin sensitivity, and blood lipids in children [12]. The authors concluded the current evidence between protein intake and these cardiovascular biomarkers to be inconclusive. In addition, a narrative review on the impact of diet on cardiovascular health revealed that results were inconsistent even for those dietary factors that were studied most, such as fast food and sugar-sweetened beverages [13]. The results of these reviews indicate that various factors such as measurement errors may exist that obscure the true associations between diet and obesity-related biomarkers in the age group of children and adolescents. We therefore decided to conduct a systematic literature review to further examine eligible studies that focus on associations between diet and 12 obesity-related biomarkers in healthy children and adolescents aged 3-18 years. We aimed to: a) expand the findings of former literature reviews, b) examine, if we can confirm the inconclusive findings of these reviews, and c) if yes, take a closer look on possible reasons for those inconclusive findings.

## Methods

### Literature search

**Fig. 1:**
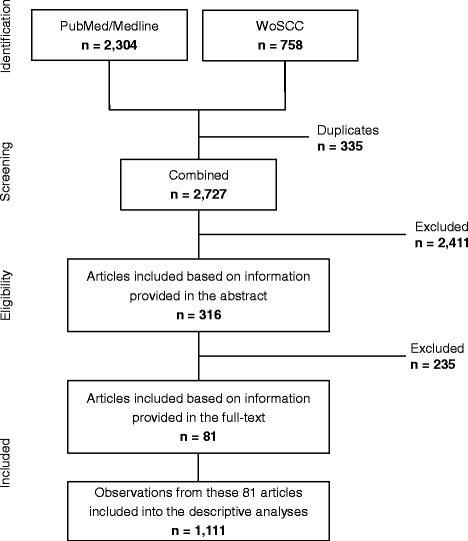
Flow diagram of the study selection process

We conducted our systematic literature review in accordance to the PRISMA Statement [14]. We searched the scientific databases *PubMed/Medline* and *Web of Science Core Collection (WoS CC)* for potentially eligible articles. We developed a systematic search strategy, which included the following Medical Subject Heading terms from PubMed: “child”, “adolescent”, “food and beverages”, “diet” “food quality”, “triglycerides”, “blood glucose”, “blood pressure”, and “C-reactive protein”. In addition, we took into account free-text terms like “dietary intake” and “biomarker”. For the *WoS CC* database we adapted our final PubMed search strategy. In Additional file 1 we provide the exact search strategies of both literature databases. The final screen on 2016/02/29 resulted in 2727 hits (Fig. 1) from both databases after excluding 335 duplicates identified via Endnote software (Thomson Reuters).

### Study selection

Table 1 provides an overview of the a priori defined inclusion and exclusion criteria that we applied to select eligible articles. The exposure variable was a dietary factor (macronutrient, single food, dietary pattern). The outcomes of interest were obesity-related biomarkers (Table 1), which we had previously derived from two existing literature reviews that investigated associations between biomarkers and obesity in children and adolescents [8, 15]. Barkin, et al. [15] revealed that biomarkers related to obesity in adults (e.g., cortisol) did not show such associations in children. We therefore took only those biomarkers into account that were associated with obesity in children.

**Table 1:** Inclusion and exclusion criteria

Study characteristics	Included	Excluded
Exposures	At least one of the following:	
	• Food or food group (e.g., biscuits, sweets) • Macronutrient (e.g., protein, carbohydrates) • Dietary pattern (e.g., high-fat dietary pattern)	• Micronutrients (e.g., vitamin A, iron, sodium) • Alcoholic beverages
Outcomes	At least one of the following:	
	• Fasting triglycerides • Total cholesterol • HDL cholesterol • LDL cholesterol • Fasting insulin • Fasting glucose • (HOMA-) insulin resistance • Insulin sensitivity • C-reactive protein • Blood pressure (systolic and/or diastolic) • Adiponectin • Leptin	• Other biomarkers
Populations	• Healthy children and/or adolescents (age range: 3-18 years) • Subgroups included within these age range (e.g., 5-10 years; 3-15 years) • Children and/ or adolescents who are overweight/obese	- Infants (age group: 0 < 3 years) - Adults (age group: > 18 years) - Patient samples (e.g., children with diabetes, asthma)
Study designs	• Longitudinal studies ○ Prospective studies ○ Cohort studies • Cross-sectional studies	• Intervention studies (e.g., randomized control trials) • Case-control studies • Reviews/ Meta analyses • Case series or case reports • Qualitative studies • Comments • Animal studies
Other criteria	• Original articles, short reports, brief reports • Studies published in English • Human studies	• Studies published in languages other than English

*HDL* High-density lipoprotein, *LDL* Low-density lipoprotein, *HOMA* Homeostatic model assessment

Three of the authors (JHK, IM, CB) independently screened all 2727 abstracts. By applying inclusion/exclusion criteria to the information contained in the abstract, we reduced the pool of potentially eligible articles to 316 (Fig. 1). The same three authors also evaluated the retrieved full-text articles applying the same inclusion and exclusion criteria that were used for the abstract selection. Any disagreements during the selection process were discussed among all three reviewers since consensus was reached. Finally we included 81 full-text articles into our review.

### Data extraction, data elements, and quality assessment

Three of the authors (JHK, IM, CB) extracted relevant information from all 81 articles using a standardized data extraction template. We designed our template with the intent of shedding light on possible reasons for inconclusive findings. Thus we included detailed information on food/nutrient intake (e.g., assessment method, nutrient databases) and biomarkers (e.g., assessment method, blood collection procedures), in addition to general information on study design and characteristics of the study population (e.g., recruitment methods, sample size). We also extracted from each article details on data analyses (e.g., statistical methods, adjustment for confounders) and relevant information to evaluate a potential risk of bias.

Study quality was assessed by two of the authors (JHK, CB) independently. We slightly adapted a quality assessment tool that was originally developed by Voortman, et al. [12] to evaluate study quality of all articles included in our review. The quality assessment tool used a rating scheme ranging from low to high (low quality: 0-4, moderate quality: 5-8, high quality: 9-11). We took into account the following characteristics to rate the study quality: study design, sample size, intake validity, adjustment for potential confounders, and the occurrence of a selection bias (Additional file 2). We decided to use this assessment tool because it takes criteria into account that are unique for nutrition studies (e.g., the validity of the dietary assessment) and thus are not reflected by the standard tools. As measurement error in dietary intake assessment might also be on potential explanation for inconclusive findings we preferred the tool introduced by Voortman et al. 2015 to quality assessment tools conventionally used in systematic reviews.

### Data preparation

Two of the authors (CB, JHK) rechecked a random sample of all data extracted to ensure high data quality. Afterwards, we prepared data for descriptive data analyses. In some cases, we extracted multiple subentries (from now on labeled as observations) from one article because they reported one value for the total study sample and, for example, additional values for either males or females. In such cases, we decided to exclude the overall value from further analyses to avoid double counting of individuals. In addition, several articles reported on associations between a dietary factor and more than one biomarker (e.g., blood pressure, total cholesterol) or on associations between different dietary factors (e.g., vegetables, dairy products, sweets) and one biomarker. In these cases, we kept multiple observations from one article in the descriptive analyses as these observations provided separate information of interest.

### Data analyses

We conducted descriptive analyses to give an overview of the main characteristics of all articles included (see Additional file 3 for a detailed overview of the characteristics of each single study). Due to heterogeneity of the articles with regard to, for example, study designs, dietary assessment methods, and statistical analyses, we decided that conducting a formal meta-analysis was not appropriate. Instead we provide a descriptive summary of our results.

## Results

We included a total of 81 articles with an overall sample size of 52,764 (sample sizes range: 79-21,111). The majority of articles (79.0%) had a cross-sectional design. Most articles assessed dietary intake using food frequency questionnaires (FFQs; 38.3%), followed by 37.0% of articles that used 24-h recalls. Following our quality assessment tool we rated more than half of the articles (58.0%) low in quality and only 3.7% achieved a high quality score (Table 2).

**Table 2:** Main characteristics of the studies included in the systematic review

Main characteristics	n articles (%)
*Sample size*	
< 500 or not reported	46 (56.8)
≥ 500 < 1000	15 (18.5)
≥ 1000	20 (24.7)
*Age group (articles can be included in more than one age category)*	
3 to 7 years	22 (27.2)
8 to 12 years	39 (48.1)
13 to 18 years	64 (79.0)
*Study design*	
Cross-sectional study	64 (79.0)
Prospective cohort study	17 (21.0)
Details on dietary assessment	
*Dietary assessment method*	
FFQ	31 (38.3)
24 h recall	30 (37.0)
Dietary record	9 (11.1)
Combination of two methods	7 (8.6)
Other	4 (5.0)
*Frequency of dietary assessments*	
Once	40 (49.4)
Twice	28 (34.6)
Three times or more	13 (16.0)
*Validity of dietary assessment (as reported by the authors)*	
Yes	31 (38.3)
No	10 (12.3)
Unknown	40 (49.4)
*Adults’ involvement in dietary assessment*	
All studies	35 of 81(43.2)
Studies with children aged 3–7 years	17 of 22 (77.2)
Studies with children aged 8–11 years	21 of 39 (53.8)
Studies with children aged 12–18 years	17 of 64 (26.6)
*Study quality*	
Low	47 (58.0)
Moderate	31 (38.3)
High	3 (3.7)

**Fig. 2:**
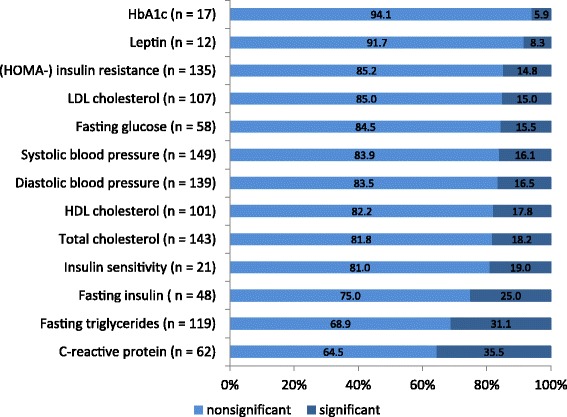
Overview of associations found for the different obesity-related biomarkers

The 81 articles reported on 1111 single observations of associations between a dietary factor and a biomarker of interest. Figure 2 shows the number of observations found for each biomarker of interest (besides Adiponectin, where no observations could be identified). We identified most observations for systolic blood pressure (*n* = 149), followed by observations on total cholesterol (*n* = 143), diastolic blood pressure (*n* = 139), and Homeostatic Model Assessment (HOMA)-insulin resistance (*n* = 135). Overall, 19.2% of all 1111 observations found significant associations between a dietary factor and any of the obesity-related biomarkers considered. Figure 2 also provides an overview on the percentage of significant associations separated by biomarker. In relative terms, we observed the highest percentage of significant associations for C-reactive protein (CRP; 35.5%) followed by fasting triglycerides (31.1%), and fasting insulin (25.0%). For all other biomarkers the number of significant associations observed was below 20.0% (Fig. 2). A separate consideration of 180 observations (18 articles) that examined associations between dietary patterns and obesity- related biomarkers revealed that 17.2% (*n* = 31) of these observations were significant.

As mentioned above we observed the highest percentage of significant associations for CRP. Table 3 provides a brief overview of all observations (*n* = 62) found between dietary factors and CRP levels. Overall, these observations came from 13 articles that reported on 31 different dietary factors. For most of these dietary factors, only one or two observations were available that reported on possible associations between CRP and the respective dietary factor. In cases where we identified more than two observations, results were inconsistent. For example, Aeberli et al. 2006 [16] found a significant association between fat and CRP levels (β = 0.28; *p* = 0.007). In contrast, Thomas et al. 2008 [17] did not find such an association, neither for girls (*r* = 0.05; *p* = 0.705) nor for boys (*r* = −0.25; *p* = 0.053). For some associations, we also observed sex differences (e.g., for whole grains [18]).

**Table 3:** Associations reported for dietary factors and C-reactive protein (*n* = 62)

First author, year, country	Sample characteristics	Study design	Dietary assessment	Validity dietary assessment	Subgroups	Dietary factor	Effect estimates	Statistical method, Effect size	Selection bias	Quality score
	Outcome: C-reactive protein									
Aeberli et al. 2006, Switzerland [16]	n: 79; age: 6-14; female: 46.8%	Cross-sectional	24 h recall; 1 day food record	Yes	no	Fat Fat (% energy) SFA PUFA MUFA Dairy products Meat Plant oils Animal fat	0.28** 0.28** 0.24* 0.21* 0.27* n.s. n.s. 0.24* n.s.	Multiple regression and ANCOVA, *β*-coefficient	Yes	3
Au et al. 2012, USA [42]	n: 148; age: 9-15; female: 58.8%	Cross-sectional	FFQ	Yes	no	SFA MUFA PUFA Carbohydrates	n.s. n.s. n.s. n.s.	Linear regression, *β*-coefficient	Yes	3
Chan et al. 2015, Australia [43]	n: 2262; age: 14, 17; female: 50.4%	Prospective cohort study	FFQ	Unknown	n: 1458	DGI-CA	n.s.	Linear regression, *β*-coefficient	Unknown	7
Gonzalez Gil et al. 2015, Multiple European countries [44]	n: 3884; age: 6-9; female: 50.9%	Cross-sectional	FFQ	No	Boys Girls	Raw vegetables Raw vegetables	0.7* n.s.	Multilevel ordinal logistic regression, Odds ratio	Unknown	5
Holt et al. 2009, USA [45]	n: 285; age: 13-17; female: 45.6%	Cross –sectional; Cohort study	FFQ	Yes	no	Fruits (no fruit juice) Fruit juice Vegetables French fries Legumes Fruits/Vegetables	−0.19** n.s. n.s. n.s. n.s. −0.15*	Spearman partial correlation coefficients, Correlation coefficient	Yes	4
Hur et al. 2012, USA [18]	n: 4928; age: 12-19; female: 49.4%	Cross-sectional	24 h recall	Unknown	Boys Girls	Whole grains Whole grains	n.s.^a^ significant^a^	Multiple linear regression, Adjusted mean values	No	8
Kosova et al. 2013, USA [46]	n: 4880; age: 3-11; female: 49.3%	Cross-sectional	24 h recall	Unknown	Age 3-5 (n: 1227)	SSB	n.s.	Linear regression, Adjusted *β*-coefficient	No	9
					Age 6-8 (n: 1316)	SSB	n.s.			
					Age 9-11 (n: 1375)	SSB	0.01*			
Lin et al. 2014, Multiple European countries [47]	n: 1804; age: 12.5-17.5; female: 52.6%	Cross-sectional	24 h recall	Yes	no	Dietary fiber Soluble Fiber Insoluble Fiber	n.s. n.s. n.s.	GLM multivariate analysis, *β*-coefficient	Unknown	6
Qureshi et al. 2009, USA [48]	n: 4110; age: 5-16; female: 49.9%	Cross-sectional	24 h recall	Unknown	no	Dairy products Milk Cheese Yoghurt Grains Refined grains Whole grains Fruits Citrus, melon, berries Other fruits Vegetables Non starchy vegetables	significant^a^ significant^a^ n.s.^a^ n.s.^a^ significant^a^ significant^a^ n.s.^a^ n.s.^a^ significant^a^ n.s.^a^ significant^a^ significant^a^	ANOVA, Mean differences	No	7
						Dark green vegetables Deep yellow/orange	n.s.^a^			
						Vegetables Tomatoes	n.s.^a^			
						Starchy vegetables Legumes Potatoes Meat/other Proteins Red meat White meat Other Proteins sources	significant^a^ significant^a^ n.s.^a^ n.s.^a^ n.s.^a^ n.s.^a^ n.s.^a^ n.s.^a^			
Thomas et al. 2008, UK [17]	n: 164; age: 12-13; female: 54.3%	Cross-sectional	FFQ, food diary	Yes	Boys Girls	Fat SFA Fat SFA	n.s. n.s. n.s. n.s.	Pearson correlation, Partial correlation coefficient	No	4
Truthmann et al. 2012, Germany [34]	n: 5198; age:12-17; female: 49.1%	Cross-sectional	FFQ	Yes	Boys (n: 2554)	HFD HuSKY IFI F & V Index HFD HuSKY IFI F & V Index	n.s. −8.20* n.s. n.s. n.s. n.s. −14.00** n.s.	Linear regression, *β*-coefficient	No	10
					Girls (n: 2438)					
Vyncke et al. 2013, Multiple European Countries [49]	n: 552; age: 12.5-17.5; female: 52.0%	Cross-sectional	24 h recall	Yes	Boys Girls	DQI-A DQI-A	n.s. n.s.	Multilevel regression models, *β*-coefficient	Yes	2
Zhu et al. 2014, USA [50]	n: 5124; age: 2-18; female: 51.3%	Cross-sectional	FFQ	Unknown	n: 3769	Yoghurt	n.s.^a^	Linear regression, Least square means	Unknown	6

ANCOVA: Analysis of Covariance; *DGI-CA* Dietary Guideline Index for Children and Adolescents, *DQI-A* Dietary Quality Index, *FFQ* Food Frequency Questionnaire, *HFD* Healthy Food Diversity Index, *HuSKY* Healthy Nutrition Score for Kids and Youth, *IFI* Indicator Food Index, *MUFA* Monounsaturated Fatty Acids, *PUFA* Polyunsaturated Fatty Acids, *SFA* Saturated Fatty Acids, *SSB* Sugar sweetened beverages

Effect estimates: *p*< 0.01 ** *p*< 0.05: *; Quality Score: 0-4: low; 5-8: moderate; 9-11: high

n.s.: not significant; n.a.: not available

^a^Categorized intake variable, please see original manuscript for further details

We also saw such inconsistencies for systolic blood pressure, the biomarker with most observations found. About 16.0% of all 149 observations identified (Fig. 2), showed a significant association between a dietary factor and systolic blood pressure. For example, we found six observations (derived from three articles: [19–21]) that examined associations between sugar sweetened beverages and systolic blood pressure. While four of these six observations showed nonsignificant results, two observations reported a positive association between sugar sweetened beverages and systolic blood pressure ([20, 21]; Additional file 3). However, while Bremer et al. 2009 [20] reported a significant association for girls only (β = 0.38; *p* < 0.05), Chan et al. 2009 [21] found such an association for boys (β = 1.6; *p* < 0.043) but not for girls (β = 0.8; *p* = 0.171). We saw a similar inconsistent picture for all other biomarkers of interest (Additional file 3).

## Discussion

### Summary of main findings and comparison with previous reviews

Our systematic review revealed that only a minority (19.2%) of the total observations showed significant associations between a dietary factor and any of the obesity-related biomarkers included. Furthermore, for many biomarkers we were not able to identify enough observations to draw clear conclusions on possible associations between a dietary factor and the respective biomarker. Our results confirm the findings of existing reviews [12, 13]. Since nonsignificant and inconclusive findings may impede the development of effective strategies against childhood obesity, we decided to take a closer look at possible reasons for these findings.

### Possible reasons for the nonsignificant and inconclusive findings in our review

#### Dietary intake

The common approach to studying diet-disease relationships focuses on single nutrients or food items. One major criticism of this approach is that typical diets do not consist of single nutrients or foods: indeed, these items are eaten in combination [22]. Another shortcoming of this approach is that the effect of a single nutrient might be too small to detect [22]. Therefore, an examination of dietary patterns has been suggested because these patterns may reflect the complexity of the diet better than single nutrients or food items [23]. In addition, Hu suggested that the cumulative effects of multiple nutrients or food items reflected by a dietary pattern might be large enough to be detected [22]. However, at least in our review, taking into account only observations on dietary patterns did not change the results.

### *Measurement errors in dietary intake assessment*

Nutrition epidemiology is also affected by bias resulting from imprecision in the measurement of dietary intake [24]. Measurement error may cause over- or underestimation of the impact of exposure [24]. Thus the accurate assessment of dietary intake in children and adolescents is essential not only to monitor nutritional status but also to draw reliable conclusions on diet-disease relationships within this age group [25]. However, valid assessment of dietary intake can be particularly difficult for this age group [26]. Children younger than 8 years do not have the cognitive abilities to report their dietary intake [27]. Therefore, involving proxy respondents like parents or other caregivers is necessary to obtain information on children’s dietary intake [27]. Overall, 43.2% of all articles included in our review reported the involvement of parents in dietary intake assessment. In the youngest age group (three- to seven-year-old children), the percentage was even higher, with more than 75% of articles stating that parents assisted in dietary intake assessment. However, parents often do not know what their children have consumed when the children are supervised by other persons, for example, by their teachers at school [28]. Another issue in the assessment of dietary intake is the estimation of portion sizes: most children, in addition to lacking the cognitive abilities necessary to accurately report portion sizes, simply do not pay attention to frequencies and portion sizes while they are eating [27]. Among adolescents valid reporting of dietary intakes is affected by unstructured eating patterns, increased out-of-home eating, and lack of motivation [27]. Another possibility of measurement error is the dietary assessment method itself. Although 24-h recalls, FFQs, and dietary records are the common methods to assess dietary intake, their accuracy and appropriateness in the age group of children has been questioned [29]. A current systematic review indicates that the FFQ might be the most appropriate method to assess dietary intake in children aged 11 years and younger [29]. However, the authors concluded that further research on the validity and reliability of dietary assessment methods in children is needed due to a limited generalizability of the results.

Moreover, current dietary assessment methods are prone to social desirability bias [13]. Underreporting of foods considered as unhealthy (e.g., sugar-sweetened beverages) and overreporting of foods perceived as healthy (e.g., fruits and vegetables) might be another reason why we do not find consistent associations between a dietary factor and the obesity-related biomarkers included. Archer et al. [30] examined the issue of dietary reporting error in different waves of the US National Health and Nutrition Examination Survey (NHANES). They found that the self-reported energy intakes were implausible for more than half of all participants [30]. Moreover, a large European multicenter study in children and infants revealed that parental underreporting was strongly affected by parental concerns/perceptions of their child’s weight status [31].

#### Biomarkers

Furthermore, it may be that inappropriate biomarkers are being measured in children. For example, there might be differences in the metabolism of nutrients between children and adults. Kostyak, et al. [32] reported that, compared to adults, pre-pubertal children oxidize greater amounts of fat per calorie expended each day. Such differences in metabolism may also exist for other nutrients, and thus the age group might be too young to observe associations between dietary intake and the biomarkers considered in our review. Another explanation might be that metabolic consequences may not occur before early adulthood because children and adolescents may cope with an unbalanced diet better than adults can. Former studies indicate that puberty seems to influence biomarker levels. For example, HDL cholesterol levels have been found to be higher among girls at pubertal stage compared to boys [33, 34]. This may explain sex differences that we observed for some of the diet-biomarker associations. In addition, it is important that these differences are taken into account in statistical analyses by conducting separate analyses for girls and boys or at least by adjusting the analyses for the pubertal stage.

### *Measurement errors at the biomarker level*

In contrast to the measurement of dietary intake, subjective reporting errors like social desirability bias are not affecting biomarkers [35]. However, biomarker measurements are not free of error. For example, blood sampling techniques, storage procedures, and laboratory assay errors can influence the results [36]. In addition, biomarker levels may vary over time within an individual; a single biomarker measurement, as common in many epidemiological studies [36], may thus not be able to reflect long-term consequences of diet. Another shortcoming of biomarker measurement is determining adequate reference values for the age group of children and adolescents [37]. For example, there are currently four methods used to determine insulin levels: bioassays, high-performance liquid chromatography, stable isotope dilution mass spectrometry assay, and immunoassays [37]. However, separate reference values for the pediatric population have not been defined for any of these methods [37]. As discussed above, growth and pubertal stage may affect biomarkers in this age group, making the establishment of reference intervals for children and adolescents a major challenge [37].

#### Study quality

Another reason why we did not find clear associations between dietary factors and the obesity-related biomarkers may be related to study quality. The majority of the studies included (58.0%) were rated low in quality, mainly because few articles considered important confounding factors like energy intake and body weight. This finding is in line with the review by Voortman, et al. [12] on protein intake and cardiovascular health.

In our review the low sample sizes reported in the majority of articles (56.8%) may not have allowed an adequate adjustment for potential confounders in most studies included. Furthermore, these low sample sizes may have resulted in a low statistical power and thus may have reduced the chance of detecting significant associations. In addition, most of the articles included in our review reported on cross-sectional studies. As long-term exposure to unbalanced diets and not eating a single unhealthy meal causes diet-related diseases, cross-sectional studies may not be able to adequately reflect the adverse health effects of unbalanced diets [38].

### Action points for the future

Longitudinal studies in particular should be conducted to obtain information on long-term consequences of dietary intake on obesity-related biomarkers in children and adolescents. In addition, large sample sizes are necessary to ensure adequate adjustment for important confounding factors such as: sex, age, energy intake and anthropometric measurements. In addition, other confounding factors include: pubertal stage, sex hormones, parental overweight, physical activity, and socioeconomic status.

The development of novel dietary assessment methods for children and adolescents or at least a refinement of existing methods is necessary and should take into account age, cognitive abilities, and adequate tools for portion size estimation [27]. In addition, tools to assess dietary intake in adolescents should be less burdensome and more able to motivate young people, as a lack of motivation in reporting dietary intake seems to exist in this age group [27]. As the age group of adolescents is very computer literate, smartphone applications for assessing dietary intake may greatly improve dietary intake reporting within that age group [27]. Furthermore, we also need better reporting on the details of dietary assessment. For example, a statement on the validity of the dietary assessment method used should be given. Moreover, it is important that novel dietary assessment methods reflect the usual diet of an individual and not merely provide a snapshot of what an individual has eaten during a single day or week. Smartphone technologies may be helpful in collecting long-term data on dietary intake without too much effort for the individual.

In addition, novel biomarkers that better reflect dietary intake in the age group of children and adolescents are necessary [35]. These biomarkers should be valid, noninvasive, cost-effective, and able to reflect changes in dietary intake over time [35]. Moreover, they should enable researchers and health professionals to detect early on children that have an increased risk of becoming overweight or obese. Kuhnle suggested to analyze hair specimens as an alternative to the current blood sample analyses, due to its ability to reflect long-term diet [39]. The emerging field of metabolomics may also be helpful in discovering novel biomarkers [35]. Current metabolomic screenings identified urinary markers that are associated with the consumption of a specific food or food groups [40]. For example, researchers found urinary markers reflecting the intake of oily fish, a meat-rich diet, and a vegetable-rich diet [40]. Furthermore, metabolomics may be helpful in validating the findings from observational or epidemiological studies in the future [41].

### Strengths and limitations

The major strengths of our literature review include the application of a systematic search strategy and adherence to PRISMA guidelines [14]. Furthermore, we searched for eligible studies in two of the most renowned biomedical literature databases, which also decreased the probability of missing relevant articles. However, as we only included peer-reviewed articles we cannot fully exclude the occurrence of a publication bias. In addition, a language bias may have affected our results as we only took English-language articles into account. Nevertheless, our literature review provides valuable insights into possible reasons for the nonsignificant and inconclusive findings with regard to associations between dietary factors and obesity-related biomarkers in the age group of children and adolescents.

## Conclusions

Our systematic review confirmed that associations between diet and obesity-related biomarkers are nonsignificant and inconclusive. We thus focused on possible reasons for these inconclusive findings because they may impede the development of effective strategies against childhood obesity. We provided action points for future research efforts, such as improving dietary intake assessment in children and adolescents and identifying appropriate obesity-related biomarkers. Such research efforts are urgently needed to clarify the role of diet in early stages of obesity development and may enable the implementation of evidence-based interventions to prevent childhood obesity.

## Additional files

Additional file 1: Detailed search strategies for Pubmed and WoS CC. (DOCX 22 kb)

Additional file 2: Quality score used to assess study quality. (DOCX 18 kb)

Additional file 3: Detailed characteristics of all 81 studies included. (DOCX 690 kb)

## Abbreviations

CRP: C-reactive protein
FFQ: Food frequency questionnaire
HOMA-IR: Homeostatic model assessment-insulin resistance
NHANES: National Health and Nutrition Examination Survey
WoS CC: Web of Science Core Collection
