# Supplementary material for: Associations between dietary factors and obesity-related biomarkers in healthy children and adolescents - a systematic review
Source: Nutr J. 2017 Dec 28;16:85. doi: 10.1186/s12937-017-0300-3 (PMC5745631; doi:10.1186/s12937-017-0300-3)
Supplement: Supplementary file 1 — Detailed search strategies for Pubmed and WoS CC. (DOCX 22 kb) [file 12937_2017_300_MOESM1_ESM.docx]

**Additional file 1: Detailed search strategies for PubMed and WoS CC**

| **PubMed/Medline  (Final screen: 2016/02/29, 2,304 hits)** | **Web of Science Core Collection**  **(Final screen: 2016/02/29, 758 hits)** |
| --- | --- |
| (  ("Child"[MeSH Terms] OR  "Adolescent"[MeSH Terms] OR  minors[tiab] OR  boy*[tiab] OR  girl*[tiab] OR  kid[tiab] OR  kids[tiab] OR  child*[tiab] OR  schoolchild*[tiab] OR  adolescen*[tiab] OR  teen*[tiab] OR  preteen*[tiab] OR  underage*[tiab] OR  under age*[tiab] OR  pubescen*[tiab] OR  juvenil*[tiab] OR  youth*[tiab])  AND  ("Food and beverages"[MeSH Terms] OR  "diet"[MeSH:noexp] OR  "energy intake"[MeSH Terms] OR  "nutrition assessment"[MeSH Terms] OR  "food habits"[MeSH Terms] OR  "food quality"[MeSH Terms] OR  "Nutritive value"[MeSH Terms] OR  food*[Title/Abstract] OR  "diet"[Title/Abstract] OR  dietary intake*[Title/Abstract] OR  energy intake*[Title/Abstract] OR  energetic intake*[Title/Abstract] OR  caloric intake*[Title/Abstract] OR  nutrition intake*[Title/Abstract] OR  nutritive intake*[Title/Abstract] OR  nutritional intake*[Title/Abstract] OR  nutrition assessment*[Title/Abstract] OR  nutritional assessment*[Title/Abstract] OR  nutrition survey*[Title/Abstract] OR  nutritional survey*[Title/Abstract] OR  nutrition index*[Title/Abstract] OR  nutritional index*[Title/Abstract] OR  "nutrition indices"[Title/Abstract] OR  "nutritional indices"[Title/Abstract] OR  nutrition value*[Title/Abstract] OR  nutritive value*[Title/Abstract] OR  nutritional value*[Title/Abstract] OR  nutrition qualit*[Title/Abstract] OR  nutritive qualit*[Title/Abstract] OR  nutritional qualit*[Title/Abstract] OR  dietary pattern*[Title/Abstract] OR  dietary habit*[Title/Abstract] OR  eating pattern*[Title/Abstract] OR  dietary fiber*[Title/Abstract] OR  dietary carbohydrate*[Title/Abstract] OR  dietary protein*[Title/Abstract] OR  dietary fat*[Title/Abstract])  AND  ("Triglycerides"[Mesh Terms] OR  "Cholesterol"[Mesh Terms] OR  "Insulin"[MeSH Terms] OR  "blood glucose"[MeSH Terms] OR  "insulin resistance"[MeSH Terms] OR  "C-Reactive Protein"[Mesh Terms] OR  “Hemoglobin A, Glycosylated"[Mesh Terms] OR  "Blood Pressure"[Mesh Terms] OR  "Adiponectin"[Mesh Terms] OR  "Leptin"[Mesh Terms] OR  biological marker*[Title/Abstract] OR  biomarker*[Title/Abstract] OR  Triglyceride*[Title/Abstract] OR  Triacylglycer*[Title/Abstract] OR  "Cholesterol"[Title/Abstract] OR  "Insulin"[Title/Abstract] OR  "blood glucose"[Title/Abstract] OR  “plasma glucose“[Title/Abstract] OR  "serum glucose“[Title/Abstract] OR  "fasting glucose”[Title/Abstract] OR  "HbA1C”[Title/Abstract] OR  "HbA 1C” [Title/Abstract] OR  "Hb A1C” [Title/Abstract] OR  "insulin resistance"[Title/Abstract] OR  “insulin sensitivity"[Title/Abstract] OR  C-Reactive Protein*[Title/Abstract] OR  “CRP"[Title/Abstract] OR  Glycosylated Hemoglobin*[Title/Abstract] OR  Glycated Hemoglobin*[Title/Abstract] OR  “HOMA-IR”[Title/Abstract] OR  "Blood Pressure"[Title/Abstract] OR  "Adiponectin"[Title/Abstract] OR  "Leptin"[Title/Abstract] OR  “ob protein”[Title/Abstract])  AND  (“epidemiologic studies"[Mesh Terms] OR  epidemiologic stud*[Title/Abstract] OR  epidemiological stud*[Title/Abstract] OR  cross sectional stud*[Title/Abstract] OR  cohort stud*[Title/Abstract] OR  longitudinal stud*[Title/Abstract] OR  longitudinal survey*[Title/Abstract] OR  prospective stud*[Title/Abstract])  )  NOT  ("nutrition therapy"[mh] OR  "pregnancy"[mh] OR  "maternal-fetal relations"[mh] OR  "Pregnant Women"[mh] OR  "therapy"[sh] OR  "Review"[Publication Type]) | TS=(  (  (child* OR  adolescen* OR  "minors" OR  boy* OR  girl* OR  "kid" OR  "kids" OR  schoolchild* OR  teen* OR  preteen* OR  underage* OR  "under age*" OR  pubescen* OR  juvenil* OR  youth*)  AND  (food* OR  "diet" OR  "dietary intake*" OR  "energ* intake*" OR  "caloric intake*" OR  "nutriti* intake*" OR  "nutriti* assessment*" OR  "nutriti* survey*" OR  "nutriti* index*" OR  "nutriti* indices" OR  "nutriti* value*" OR  "nutriti* qualit*" OR  "dietary pattern*" OR  "dietary habit*" OR  "eating pattern*" OR  "dietary fiber*" OR  "dietary carbohydrate*" OR  "dietary protein*" OR  "dietary fat*")  AND    ("biological marker*" OR  Biomarker* OR  Triglyceride* OR  Triacylglycer* OR  "Cholesterol" OR  "Insulin" OR  "blood glucose" OR  "plasma glucose" OR  "serum glucose" OR  "fasting glucose" OR  "HbA1C" OR  "HbA 1C" OR  "Hb A1C" OR  "insulin resistance" OR  "insulin sensitivity" OR  "C-Reactive Protein*" OR  "CRP" OR  "Glycosylated Hemoglobin*" OR  "Glycated Hemoglobin*" OR  "HOMA-IR" OR  "Blood Pressure" OR  "Adiponectin" OR  "Leptin" OR  "ob protein")  AND  (adipos* OR  obes* OR  "overweight" OR  "body weight" OR  "bodyweight" OR  "body mass index" OR  "bodymass index" OR  "skinfold thickness*" OR  "waist circumference*" OR  "waist hip")  AND  ("epidemiologic* stud*" OR  "cross sectional stud*" OR  "cohort stud*" OR  "longitudinal stud*" OR  "longitudinal survey*" OR  "prospective stud*")  )  NOT  ("nutrition therapy" OR  pregnan*)  ) |
|  | FILTER reviews |
