# Supplementary material for: Associations between dietary factors and obesity-related biomarkers in healthy children and adolescents - a systematic review
Source: Nutr J. 2017 Dec 28;16:85. doi: 10.1186/s12937-017-0300-3 (PMC5745631; doi:10.1186/s12937-017-0300-3)
Supplement: Supplementary file 2 — Quality score used to assess study quality. (DOCX 18 kb) [file 12937_2017_300_MOESM2_ESM.docx]

**Additional file 2: Quality score to assess study quality**

Study design

**0** for studies with cross-sectional data analysis

**1** for studies with longitudinal data analysis

Populations (2x)

**0** if n < 500 OR if not reported

**1** if n ≥ 500 and < 1000

**2** if n ≥ 1000

Exposure / Influence factor

**0** if the study used no appropriate dietary assessment method OR if not reported

**1** if the study used a *“one day food record”*, *“one 24 hour recall”*, a *“short food frequency questionnaire (< 50 food items)”* AND no statement about the validity of the method was made

**2** if the study used *“multiple day food records”*, “multiple 24 hour recalls”, a full diet food frequency questionnaire (≥ 50 food items) OR a statement was made by the authors that the method used was valid

Adjustment

**0** if the analysis was not controlled for the four covariates mentioned below (under point 1) OR if not reported

**1** if findings were controlled for:

- age or Tanner stage
- sex
- energy intake (including % of energy)
- at least one measure of body weight (e.g. BMI, waist circumferences, body weight or body

fat)

**2** if findings were additionally controlled for at least two of the following covariates:

- intake of other nutrients / foods (e.g. other macronutrients, micronutrients)
- physical activity
- sedentary behaviour
- growth
- birth characteristics (birth weight, gestational age)
- maternal characteristics (e.g. maternal BMI)
- socioeconomic status (e.g. family income, parental income, parental education)
- ethnicity

Selection bias (2x)

**0** if selection bias is present or if no information about selection bias is reported

**1** if no selection bias is present

High score: 11

Low quality: 0-4

Moderate quality: 5-8

High quality: 9-11
