# Supplementary material for: Associations between dietary factors and obesity-related biomarkers in healthy children and adolescents - a systematic review
Source: Nutr J. 2017 Dec 28;16:85. doi: 10.1186/s12937-017-0300-3 (PMC5745631; doi:10.1186/s12937-017-0300-3)
Supplement: Supplementary file 3 — Detailed characteristics of all 81 studies included. (DOCX 690 kb) [file 12937_2017_300_MOESM3_ESM.docx]

**Additional file 3: Detailed characteristics on all 81 studies included**

| **First author, year, country** | **Sample characteristics** | **Study design** | **Dietary assess-ment** | **Validity dietary assessment** | **Sub-groups** | **Dietary factor** | **Effect estimates** | | **Statistical method,**  **Effect size** | **Selection bias** | **Quality Score** |
| --- | --- | --- | --- | --- | --- | --- | --- | --- | --- | --- | --- |
|  | **Outcome: Fasting triglycerides** | | | | | | | | | | |
| Ambrosini et al. 2010, Australia[1] | n: 1139;  age: 14;  female: 47.9% | Cross- sectional; Cohort study | FFQ | Unknown | Boys  Girls | “Healthy dietary pattern”  “Western dietary pattern”  “Healthy dietary pattern”  “Western dietary pattern” | n.s.^a^  n.s.^a^  n.s.^a^  n.s.^a^ | ANOVA,  Mean | | No | 9 |
| Ambrosini et al. 2013, Australia[2] | n: 1366;  age: 14-17;  female: 48.3% | Prospective cohort study | FFQ | Yes | Boys  Girls | SSB  SSB | n.s.^a^  n.s.^a^ | Mixed linear regression models,  % of change | | Unknown | 7 |
| Appannah et al. 2015, Australia[3] | n: 1163;  age: 14, 17;  female: 48.0% | Prospective cohort study | FFQ | Yes | Boys  Girls | “Energy dense, high fat and low fiber dietary pattern”  “Energy dense, high fat and low fiber dietary pattern” | n.s.  n.s. | GEE,  $\beta$-coefficient | | Yes | 6 |
| Au et al. 2012, USA[4] | n: 148;  age: 9-15;  female: 58.8% | Cross-sectional | FFQ | Yes | no | SFA  MUFA  PUFA  Carbohydrates | n.s.  n.s.  n.s.  n.s. | Linear regression,  $\beta$-coefficient | | Yes | 3 |
| Bel-Serrat et al. 2014a,  Multiple European Countries[5] | n: 454;  age: 12.5-17.5;  female: 56.0% | Cross-sectional | 24h recall | Yes | no | Proteins  Carbohydrates  Fat | n.s.  n.s.  -0.32** | Multi-level regression,  $\beta$-coefficient | | Unknown | 4 |
| Bel-Serrat et al. 2014b,  Multiple European countries[6] | n: 454;  age: 12.5-17.5;  female: 56.0% | Cross - sectional | 24h recall | Yes | Boys  Girls | Alanine  Glycine  Isoleucine  Leucine  Valine  Phenylalanine  Tryptophan  Tyrosine  Arginine  Histidine  Lysine  Asparagine acids  Glutamic acid  Serine  Threonine  Cysteine  Methionine  Proline  Alanine  Glycine  Isoleucine  Leucine  Valine  Phenylalanine  Tryptophan  Tyrosine  Arginine  Histidine  Lysine  Asparagine acids  Glutamic acid  Serine  Threonine  Cysteine  Methionine  Proline | -0.26*  -0.29*  n.s.  n.s.  n.s.  n.s.  n.s.  n.s.  -0.29*  -0.28*  -0.26*  -0.29*  n.s.  -0.30*  n.s.  n.s.  n.s.  n.s.  -0.23*  -0.23*  -0.30**  -0.29*  -0.30**  -0.30*  -0.29*  -0.30**  -0.27*  -0.25*  -0.23*  -0.23*  -0.31*  -0.31*  -0.27*  -0.29*  -0.27*  -0.29* | Multilevel linear regression,  $\beta$-coefficient | | Yes | 4 |
| Bremer et al. 2009, USA[7] | n: 6967;  age: 12-19;  female: 48.9% | Cross-sectional | 24h recall | Unknown | Boys  Girls | SSB  SSB | 0.47*  2.25* | Linear regression,  $\beta$-coefficient | | No | 7 |
| Casazza et al. 2009b, USA[8] | n: 202;  age: 7-12;  female:47.03% | Cross-sectional | 24h recall | Unknown | no | Fat  Carbohydrates  Proteins | -0.20*  0.18*  n.s. | Multiple linear regression, Standardized  $\beta$-coefficient | | Yes | 3 |
| Chan et al. 2014a, China[9] | n: 200;  age: 12-16;  female: 51.0% | Cross-sectional | FFQ | Unknown | no | SSB | significant ^a^ | Multiple linear regression, Multivariate adjusted difference | | Unknown | 1 |
| Chan et al. 2014b, China[10] | n: 2727;  age: 12-16;  female: 51.3% | Cross-sectional | FFQ | Unknown | Boys  Girls | SSB  SSB | n.s.  n.s. | Linear regression,  $\beta$-coefficient | | No | 6 |
| Chan et al. 2015, Australia  [11] | n: 2262;  age: 14, 17;  female: 50.4% | Prospective cohort study | FFQ | Unknown | n: 1512 | DGI-CA | -0.003* | Linear regression,  $\beta$-coefficient | | Unknown | 7 |
| Day et al. 2009, USA[12] | n: 489;  age: 8, 11, 14;  female: 51.3% | Cross-sectional | FFQ | Yes | Boys  Girls | Fat  Fat | n.s. ^a^  n.s. ^a^ | ANCOVA,  Mean | | Yes | 2 |
| Hong et al. 2009, South Korea[13] | n: 246;  age: 12-13;  female: 47.6% | Cross-sectional | Dietary record | Unknown | no | Carbohydrates  Proteins  Fat | n.s.  n.s.  n.s. | Partial correlation analysis, Partial correlation coefficient | | Unknown | 2 |
| Hur et al. 2012, USA[14] | n: 4928;  age: 12-19;  female: 49.4% | Cross-sectional | 24h recall | Unknown | Boys  Girls | Whole grains  Whole grains | n.s. ^a^  n.s. ^a^ | Multiple linear regression, Adjusted mean values | | No | 8 |
| Kell et al. 2014, USA[15] | n: 320;  age: 7-12;  female: 46.9% | Cross-sectional | 24h recall | Unknown | no | Added sugars  Fat | 0.11*  n.s. | Linear regression,  $\beta$-coefficient | | Unknown | 4 |
| Kosova et al. 2013, USA[16] | n: 4880;  age: 3-11;  female: 49.3% | Cross-sectional | 24h recall | Unknown | Age 3-5  (n:468)  Age 6-8  (n:560)  Age 9-11  (n:576) | SSB  SSB  SSB | n.s.  n.s.  n.s. | Linear regression, Adjusted  $\beta$-coefficient | | No | 9 |
| Kuzawa et al. 2003, Philippines[17] | n: 608  age: 14-16;  female: 50.7% | Prospective cohort study | 24h recall | Unknown | Boys  Girls | Fat  Fat | n.s.  n.s. | Partial correlation coefficiens, Partial correlation coefficient | | Yes | 3 |
| Lin et al. 2014, Multiple European countries[18] | n: 1804;  age: 12.5-17.5;  female: 52.6% | Cross-sectional | 24h recall | Yes | no | Fiber  Soluble fiber  Insoluble fiber | n.s.  n.s.  n.s. | GLM multivariate analysis,  $\beta$-coefficient | | Unknown | 6 |
| Michels et al. 2015, Multiple European countries[19] | n: 387;  age: 12.5-17.5;  female: n.a. | Cross-sectional | FFQ | No | no | Ready to eat cereals | n.s. ^a^ | Linear regression, Estimated marginal means | | Yes | 1 |
| Nobre et al. 2013, Brazil[20] | n: 227;  age: 4-5;  female: n.a. | Cross-sectional | FFQ | No | no | “Mixed dietary pattern“ (representing a typical Brazilian diet) | n.s. ^a^ | Multivariate poisson regression, Adjusted prevalence ratios | | Unknown | 1 |
| Ochoa-Avilés et al. 2014, Euqador[21] | n: 334;  age: 10-16;  female: n.a. | Cross-sectional | 24h recall | Unknown | no | “Rice-rich non-animal fat pattern”  “Wheat-dense animal-fat pattern” | n.s.  n.s. | Linear regression,  $\beta$-% | | Unknown | 2 |
| Rinaldi et al. 2012, Brazil[22] | n: 147;  age: 4-11 female: 51.7% | Cross-sectional | 24h recall | Yes | no | Dairy products  (full fat)  Carbohydrates  Proteins  SFA  Fat  MUFA  PUFA  Cholesterol  Fiber  Cereals  Meat  Legumes  Vegetables  Fruits  Sugar, sweet food  Oils and fats | n.s.  n.s.  n.s.  n.s.  0.17*  n.s.  n.s.  n.s.  n.s.  n.s.  n.s.  n.s.  n.s.  n.s.  n.s.  n.s. | Multiple linear regression,  $\beta$-coefficient | | Yes | 3 |
| Royo-Bordonada et al. 2003, Spain[23] | n: 1112;  age: 6-7;  female: 49.9% | Cross-sectional | FFQ | Unknown | no | “Dietary variety index” | n.s. | Partial correlation analysis,  Partial correlation coefficient | | No | 7 |
| Royo-Bordonada et al. 2006, Spain[24] | n: 1112;  age: 6-7;  female: 49.9% | Cross-sectional | FFQ | Unknown | no | SFA | n.s. ^a^ | T-test,  Mean | | Yes | 7 |
| Sanchez-Bayle et al. 2008, Spain[25] | n: 673;  age: 6;  female: 47.7% | Cross-sectional | 24h recall | Unknown | no | Fat  SFA  MUFA  PUFA  Carbohydrates  Proteins | positive** ^a^  n.s. ^a^  n.s. ^a^  n.s. ^a^  negative* ^a^  n.s. ^a^ | ANOVA,  Differences in means | | Unknown | 3 |
| Scaglioni et al. 2004, Italy[26] | n: 105;  age: 8;  female: 41.0% | Cross-sectional | FFQ;  24h recall | Unknown | no | High pasta, low red meat vs. Low pasta, high red meat | n.s. | Mann-Whitney U test, Mean | | Unknown | 2 |
| Shang et al. 2012, China[27] | n: 6974;  age: 6-13;  female: 49.0% | Cross-sectional | FFQ;  24h recall | No | no | SSB vs. milk, vs. other beverages | n.s.^a^ | General linear model, Mean differences | | Unknown | 6 |
| Song et al. 2015, Korea[28] | n: 2209;  age: 10-18;  female: 47.3% | Cross-sectional | 24h recall | Unknown | Boys  Girls | Carbohydrates  White rice  Carbohydrates  White rice | n.s. ^a^  n.s. ^a^  n.s. ^a^  n.s. ^a^ | Multivariate linear regression,  Quartiles | | No | 8 |
| Steffen et al. 2003, USA[29] | n: 285;  age: 13, 15;  female: 45.6% | Prospective cohort study | FFQ | Unknown | no | Whole grains | n.s. ^a^ | Multiple linear regression, Adjusted mean values | | Yes | 4 |
| Takada et al. 1998, Japan[30] | n: 457;  age: 10;  female: 45.1% | Cross-sectional | FFQ | No | no | “Japanese diet score” | n.s. | Multiple linear regression,  $\beta$-coefficient | | Unknown | 0 |
| Van Rompay et al. 2015, USA[31] | n: 613;  age: 8-15;  female: n.a. | Cross-sectional | FFQ | No | no | SSB | n.s. ^a^ | Linear regression and ANCOVA,  Adjusted least square means | | Yes | 6 |
| Vyncke et al. 2013, Multiple European Countries[32] | n: 552;  age: 12.5-17.5;  female: 52.0% | Cross-sectional | 24h recall | Yes | Boys  Girls | DQI-A  DQI-A | n.s.  n.s. | Multilevel regression models,  $\beta$-coefficient | | Yes | 2 |
| Wajid et al. 1995, Kashmir  [33] | n: 314;  age: 5-14;  female: 46.2% | Cross-sectional | Dietary history | Unknown | no | Fat | n.s. ^a^ | Pearson correlation,  Mean | | Unknown | 1 |
| Wang et al. 2013, Canada[34] | n: 548;  age: 8-10;  female: n.a. | Cross-sectional | 24h recall | Yes | no | SSB | n.s. | Multivariate linear regression analysis,  $\beta$-coefficient | | Yes | 4 |
| Washi & Ageib 2010, Saudi Arabia[35] | n: 239;  age: 13-18;  female: 53.1% | Cross-sectional | FFQ | Unknown | no | Carbohydrates  SFA | 8.76*  7.27* | Chi-2-Test,  Chi-2-Value | | Yes | 2 |
| Zhu et al. 2014, USA[36] | n: 5124;  age: 2-18;  female: 51.2% | Cross-sectional | FFQ | Unknown | n: 1266 | Yoghurt | n.s. ^a^ | Multivariate linear regression,  Least square means | | Unknown | 6 |
|  | **Outcome: Total cholesterol** | | | | | | | | | | |
| Akerblom et al. 1984, Finland[37] | n: 233;  age: 12;  female: 46.4% | Cross-sectional | 24h recall | No | no | FA | positive** ^a^ | T-tests,  Mean difference | | No | 3 |
| Altwaiji et al. 2009, USA[38] | n: 678;  age: 8-18;  female: 49.1% | Prospective cohort study | FFQ | Yes | Boys  Girls | Cholesterol  Cholesterol | 0.01*  n.s. | Multilevel regression models,  $\beta$-coefficient | | Yes | 3 |
| Ambrosini et al. 2010, Australia[1] | n: 1139;  age: 14;  female: 47.9% | Cross –sectional; Cohort study | FFQ | Unknown | Boys  Girls | “Healthy dietary pattern”  “Western dietary pattern”  “Healthy dietary pattern”  “Western dietary pattern” | n.s. ^a^  n.s. ^a^  n.s. ^a^  positive* ^a^ | ANOVA,  Mean | | No | 9 |
| Beck et al. 2014, Brazil[39] | n: 660;  age: 14-19;  female: 52.0% | Cross-sectional | 24h recall | No | no | Lipids  SFA  Cholesterol  Fiber | n.s.  n.s.  n.s.  n.s. | Linear and multiple regression,  $\beta$-coefficient | | No | 7 |
| Bel-Serrat et al. 2014a,  Multiple European Countries[5] | n: 454;  age: 12.5-17.5;  female: 56.0% | Cross-sectional | 24h recall | Yes | no | Proteins  Carbohydrates  Fat | n.s.  n.s.  n.s. | Multi-level regression,  $\beta$-coefficient | | Unknown | 4 |
| Bel-Serrat et al. 2014b,  Multiple European countries[6] | n: 454;  age: 12.5-17.5;  female: 56.0% | Cross-sectional | 24h recall | Yes | Boys  Girls | Alanine  Glycine  Isoleucine  Leucine  Valine  Phenylalanine  Tryptophan  Tyrosine  Arginine  Histidine  Lysine  Asparagine acids  Glutamic acid  Serine  Threonine  Cysteine  Methionine  Proline  Alanine  Glycine  Isoleucine  Leucine  Valine  Phenylalanine  Tryptophan  Tyrosine  Arginine  Histidine  Lysine  Asparagine acids  Glutamic acid  Serine  Threonine  Cysteine  Methionine  Proline | n.s.  n.s.  n.s.  n.s.  n.s.  n.s.  n.s.  n.s.  n.s.  n.s.  n.s.  n.s.  n.s.  n.s.  n.s.  n.s.  n.s.  n.s.  -0.10*  -0.10*  n.s.  n.s.  -0.10*  n.s.  n.s.  n.s.  -0.11*  -0.09*  -0.08*  -0.10*  n.s.  n.s.  -0.10*  -0.11*  n.s.  n.s. | Multilevel linear regression,  $\beta$-coefficient | | Yes | 4 |
| Boulton et al. 1995, Australia  [40] | n: 134  age: 1, 2, 8, 11, 13, 15;  female: 42.5% | Prospective study | Dietary record | Unknown | Boys  Girls | Proteins  Sugar  Starch  Fat  SFA  MUFA  PUFA  Fiber  Proteins  Sugar  Starch  Fat  SFA  MUFA  PUFA  Fiber | n.s.  n.s.  n.s.  -0.24*  n.s.  -0.27*  n.s.  n.s.  n.s.  n.s.  n.s.  n.s.  n.s.  n.s.  n.s.  n.s. | Correlation analysis, Correlation coefficient | | Yes | 3 |
| Bremer et al. 2009, USA[7] | n: 6967;  age: 12-19;  female: 48.9% | Cross-sectional | 24h recall | Unknown | Boys  Girls | SSB  SSB | n.s.  n.s. | Linear regression,  $\beta$-coefficient | | No | 7 |
| Chan et al. 2014a, China[9] | n: 200;  age: 12-16;  female: 51.0% | Cross-sectional | FFQ | Unknown | no | SSB | n.s. ^a^ | Multiple linear regression, Multivariate adjusted difference | | Unknown | 1 |
| Chan et al. 2014b, China[10] | n: 2727;  age: 12-16;  female: 51.3% | Cross-sectional | FFQ | Unknown | Boys  Girls | SSB  SSB | n.s.  n.s. | Linear regression,  $\beta$-coefficient | | No | 6 |
| Chan et al. 2015, Australia[11] | n: 2262;  age: 14; 17  female: 50.4% | Prospective cohort study | FFQ | Unknown | n: 1479 | DGI-CA | n.s. | Linear regression,  $\beta$-coefficient | | Unknown | 7 |
| Day et al. 2009, USA[12] | n: 489;  age: 8, 11, 14;  female: 51.3% | Cross-sectional | FFQ | Yes | Boys  Girls | Fat  Fat | n.s. ^a^  n.s. ^a^ | ANCOVA,  Mean | | Yes | 2 |
| Franko et al. 2010, USA[41] | n: 2371;  age: 9-19;  female: 100.0% | Prospective cohort study | Dietary record | Yes | Girls | Cereals | negative* ^a^ | Mixed regression models,  Mean | | Yes | 8 |
| Fukushima et al. 1999, Japan[42] | n: 514;  age: 10-15;  female: 49.2% | Cross-sectional | FFQ | Unknown | no | Fish  Soybean  Meat  Milk  Eggs  Vegetables  Seaweed  Oil | n.s.  n.s.  n.s.  n.s.  n.s.  0.24*  n.s.  n.s. | Correlation analysis,  Correlation coefficient | | Unknown | 3 |
| Hong et al. 2009, South Korea[13] | n: 246;  age: 12-13;  female: 47.6% | Cross-sectional | Dietary record | Unknown | no | Carbohydrates  Proteins  Fat | n.s.  n.s.  n.s. | Partial correlation analysis, Partial correlation coefficient | | Unknown | 2 |
| Hur et al. 2012, USA[14] | n: 4928;  age: 12-19;  female:49.4% | Cross-sectional | 24h recall | Unknown | Boys  Girls | Whole grains  Whole grains | n.s. ^a^  n.s. ^a^ | Multiple linear regression, Adjusted mean values | | No | 8 |
| Kell et al. 2014, USA[15] | n: 320;  age: 7-12;  female: 46.9% | Cross-sectional | 24h recall | Unknown | no | Added sugars  Dietary fat | n.s.  n.s. | Linear regression,  $\beta$-coefficient | | Unknown | 4 |
| Kosova et al. 2013, USA[16] | n: 4880;  age: 3-11;  female: 49.3% | Cross-sectional | 24h recall | Unknown | Age 3-5  (n: 1153)  Age 6-8  (n: 1284)  Age 9-11  (n: 1355) | SSB  SSB  SSB | n.s.  n.s.  n.s. | Linear regression, Adjusted  $\beta$-coefficient | | No | 9 |
| Kuzawa et al. 2003, Philippines  [17] | n:608  age: 14-16;  female: 50.66% | Prospective cohort study | 24h recall | Unknown | Boys  Girls | Fat  Fat | n.s.  n.s. | Partial correlation coefficient | | Yes | 3 |
| Lin et al. 2014, Multiple European countries[18] | n: 1804;  age: 12.5-17.5;  female: 52.6% | Cross-sectional | 24h recall | Yes | no | Fiber  Soluble fiber  Insoluble fiber | n.s.  n.s.  n.s. | GLM multivariate analysis,  $\beta$-coefficient | | Unknown | 6 |
| Llyod et al. 1998, USA[43] | n: 86;  age: 16.5-17.5;  female: 100.0% | Cross-sectional | Dietary record | Unknown | Girls | Fruits | n.s. | Multiple regression,  $\beta$-coefficient | | No | 4 |
| Ochoa-Avilés et al. 2014, Euqador[21] | n: 334;  age:10-16;  female: n.a. | Cross-sectional | 24h recall |  | Rural  (n: n.a.)  Urban  (n: n.a.) | “Rice-rich non-animal fat pattern”  “Wheat-dense animal-fat pattern”  “Wheat-dense animal-fat pattern” | n.s.  3.7*  n.s. | Linear regression,  $\beta$-% | |  | 2 |
| Rinaldi et al. 2012, Brazil[22] | n: 147;  age: 4-11 female: 51.7% | Cross-sectional | 24h recall | Yes | no | Dairy products  (full fat)  Carbohydrates  Proteins  SFA  Fat  MUFA  PUFA  Cholesterol  Fiber  Cereals  Meat  Legumes  Vegetables  Fruits  Sugar, sweet food  Oils and fats | 0.36*  n.s.  n.s.  n.s.  n.s.  n.s.  n.s.  n.s.  n.s.  n.s.  n.s.  n.s.  n.s.  n.s.  n.s.  n.s. | Multiple linear regression,  $\beta$-coefficient | | Yes | 3 |
| Royo-Bordonada et al. 2003, Spain[23] | n: 1112;  age: 6-7;  female: 49.9% | Cross-sectional | FFQ | Unknown | no | “Dietary variety index” | n.s. | Partial correlation analysis,  Partial correlation coefficient | | No | 7 |
| Royo-Bordonada et al. 2006, Spain  [24] | n: 1112;  age: 6-7;  female: 49.9% | Cross-sectional | FFQ | Unknown | no | SFA | n.s. ^a^ | T-test,  Mean | | Yes | 5 |
| Sanchez-Bayle et al. 2008, Spain[25] | n: 673;  age: 6;  female: 47.7% | Cross-sectional | 24h recall | Unknown | no | Fat  SFA  MUFA  PUFA  Carbohydrates  Proteins | positive*** ^a^  positive*** ^a^  positive*** ^a^  n.s. ^a^  positive*** ^a^  n.s. ^a^ | ANOVA,  Differences in means | | Unknown | 3 |
| Scaglioni et al. 2004, Italy[26] | n: 105;  age: 8;  female: 41.0% | Cross-sectional | FFQ;  24h recall | Unknown | no | High pasta, low red meat vs. Low pasta, high red meat | n.s. | Mann-Whitney U test, Mean | | Unknown | 2 |
| Shang et al. 2012, China[27] | n: 6974;  age: 6-13;  female: 49.0% | Cross-sectional | FFQ, 24h recall | No | no | SSB; vs. milk vs. other beverages | n.s.^a^ | General linear model, Mean differences | | Unknown | 6 |
| Steffen et al. 2003, USA[29] | n: 285;  age: 13, 15;  female: 45.6% | Prospective cohort study | FFQ | Unknown | no | Whole grains | n.s. ^a^ | Multiple linear regression, Adjusted mean values | | Yes | 4 |
| Takada et al. 1998, Japan[30] | n: 457;  age: 10;  female: 45.1% | Cross-sectional | FFQ | No | no | “Japanese diet score” | n.s. | Multiple linear regression,  $\beta$-coefficient | | Unknown | 0 |
| Truthmann et al. 2012, Germany[44] | n: 5198;  age: 12-17;  female: 49.1% | Cross-sectional | FFQ | Yes | Boys  (n: 2634)  Girls  (n: 2104) | HFD  HuSKY  IFI  F &V Index  HFD  HuSKY  IFI  F & V Index | n.s.  n.s.  n.s.  n.s.  n.s.  n.s.  n.s.  n.s. | Linear regression,  $\beta$-coefficient | | No | 10 |
| Vyncke et al. 2013, Multiple European Countries[32] | n: 552;  age: 12.5-17.5;  female: 52.0% | Cross-sectional | 24h recall | Yes | Boys  Girls | DQI-A  DQI-A | -0.00061*  n.s. | Multilevel regression models,  $\beta$-coefficient | | Yes | 2 |
| Wajid et al. 1995, Kashmir  [33] | n: 314;  age: 5-14;  female: 46.2% | Cross-sectional | Dietary history | Unknown | no | Fat | positive* ^a^ | Pearson correlation, Mean | | Unknown | 1 |
| Washi & Ageib 2010, Saudi Arabia[35] | n: 239;  age: 13-18;  female: 53.1% | Cross-sectional | FFQ | Unknown | no | Carbohydrates | 6.82* | Chi-2-Test,  Chi-2-Value | | Yes | 2 |
| Zhu et al. 2014, USA[36] | n: 5124;  age: 2-18;  female: 51.2% | Cross-sectional | FFQ | Unknown | n: 3272 | Yoghurt | n.s. ^a^ | Linear regression,  Least square means | | Unknown | 6 |
|  | **Outcome: HDL-cholesterol** | | | | | | | | | | |
| Akerblom et al. 1984, Finland[37] | n: 233;  age: 12;  female: 46.4% | Cross-sectional | 24h recall | No | no | SFA  Sucrose | positive ** ^a^  negative***^a^ | T-tests,  Mean difference | | No | 3 |
| Ambrosini et al. 2010, Australia[1] | n: 1139;  age: 14;  female: 47.9% | Cross-sectional | FFQ | Unknown | Boys  Girls | “Healthy dietary pattern”  “Western dietary pattern”  “Healthy dietary pattern”  “Western dietary pattern” | positive * ^a^  n.s. ^a^  n.s. ^a^  n.s. ^a^ | ANOVA,  Mean | | No | 9 |
| Ambrosini et al. 2013, Australia[2] | n: 1366;  age: 14-17;  female: 48.3% | Prospective cohort study | FFQ | Yes | Boys  (n:587)  Girls  (n:537) | SSB  SSB | n.s. ^a^  n.s. ^a^ | Mixed linear regression,  % of change | | Unknown | 7 |
| Appannah et al. 2015, Australia[3] | n: 1163;  age: 14;17;  female: 48.0% | Prospective cohort study | FFQ | Yes | Boys  Girls | “Energy dense, high fat and low fibre dietary pattern”  “Energy dense, high fat and low fibre dietary pattern” | n.s.  0.02* | GEE,  $\beta$-coefficient | | Yes | 6 |
| Au et al. 2012, USA[4] | n: 148;  age: 9-15;  female: 58.8% | Cross-sectional | FFQ | Yes | no | SFA  MUFA  PUFA  Carbohydrates | n.s.  n.s.  n.s.  n.s. | Linear regression,  $\beta$-coefficient | | Yes | 3 |
| Beck et al. 2014, Brazil[39] | n: 660;  age: 14-19;  female:52.0% | Cross-sectional | 24h recall | No | no | Lipids  SFA  Cholesterol  Fiber | n.s.  n.s.  n.s.  n.s. | Linear and multiple regression,  $\beta$-coefficient | | No | 7 |
| Bel-Serrat et al. 2014a,  Multiple European Countries[5] | n: 454;  age: 12.5-17.5;  female: 56.0% | Cross-sectional | 24h recall | Yes | no | Proteins  Carbohydrates  Fat | n.s.  -0.19**  n.s. | Multi-level regression,  $\beta$-coefficient | | Unknown | 4 |
| Bremer et al. 2009, USA[7] | n: 6967;  age: 12-19;  female: 48.9% | Cross-sectional | 24h recall | Unknown | Boys  Girls | SSB  SSB | -0.35*  -0.73* | Linear regression,  $\beta$-coefficient | | No | 7 |
| Boreham et al. 1999, Northern Ireland[45] | n: 454;  age: 12-15;  female: 50.7% | Longitudinal cohort study | Dietary history | Unknown | Girls | Carbohydrates  Cholesterol  Fat | -0.22*  -0.09*  -0.21* | GEE,  $\beta$-coefficient | | No | 4 |
| Casazza et al. 2009b, USA[8] | n: 202;  age: 7-12;  female: 47.0% | Cross-sectional | 24h recall | Unknown | no | Fat  Carbohydrates  Proteins | n.s.  n.s.  n.s. | Linear regression, Standardized  $\beta$-coefficient | | Yes | 3 |
| Chan et al. 2014a, China[9] | n: 200;  age: 12-16;  female: 51.0% | Cross-sectional | FFQ | Unknown | no | SSB | n.s. ^a^ | Multiple linear regression, Multivariate adjusted difference | | Unknown | 1 |
| Chan et al. 2014b, China[10] | n: 2727;  age: 12-16;  female: 51.3% | Cross-sectional | FFQ | Unknown | Boys  Girls | SSB  SSB | n.s.  n.s. | Linear regression,  $\beta$-coefficient | | No | 6 |
| Chan et al. 2015, Australia  [11] | n: 2262;  age: 14, 17;  female: 50.4% | Prospective cohort study | FFQ | Unknown | n: 1479 | DGI-CA | n.s. | Linear regression,  $\beta$-coefficient | | Unknown | 7 |
| Day et al. 2009, USA[12] | n: 489;  age: 8, 11, 14;  female: 51.3% | Cross-sectional | FFQ | Yes | Boys  Girls | Fat  Fat | n.s. ^a^  n.s. ^a^ | ANCOVA,  Mean | | Yes | 2 |
| Franko et al. 2010, USA[41] | n: 2371;  age: 9-19;  female: 100.0% | Prospective cohort study | Dietary record | Yes | Girls | Cereals | n.s. ^a^ | Mixed regression models,  Mean | | Yes | 8 |
| Hong et al. 2009, South Korea[13] | n: 246;  age: 12-13;  female: 47.6% | Cross-sectional | Dietary record | Unknown | no | Carbohydrates  Proteins  Fat | n.s.  n.s.  n.s. | Partial correlation analysis, Partial correlation coefficient | | Unknown | 2 |
| Hur et al. 2012, USA[14] | n: 4928;  age: 12-19;  female: 49.4% | Cross-sectional | 24h recall | Unknown | Boys  Girls | Whole grains  Whole grains | n.s. ^a^  n.s. ^a^ | Multiple linear regression, Adjusted mean values | | No | 8 |
| Kell et al. 2014, USA[15] | n:320;  age: 7-12;  female: 46.9% | Cross-sectional | 24h recall | Unknown | no | Added sugars  Fat | n.s.  n.s. | Linear regression,  $\beta$-coefficient | | Unknown | 4 |
| Kosova et al. 2013, USA[16] | n: 4880;  age: 3-11;  female: 49.3% | Cross-sectional | 24h recall | Unknown | Age 3-5  (n: 1151)  Age 6-8  (n: 1284)  Age 9-11  (n: 1354) | SSB  SSB  SSB | n.s.  n.s.  -0.95*** | Linear regression, Adjusted  $\beta$-coefficient | | No | 9 |
| Kuzawa et al. 2003, Philippines  [17] | n: 608  age: 14-16;  female: 50.6% | Prospective cohort study | 24h recall | Unknown | Boys  Girls | Fat  Fat | n.s.  0.13* | Partial correlation coefficiens | | Yes | 3 |
| Lin et al. 2014, Multiple European countries[18] | n:1804;  age: 12.5-17.5;  female: 52.6% | Cross-sectional | 24h recall | Yes | no | Fiber  Soluble fiber  Insoluble fiber | n.s.  n.s.  n.s. | GLM multivariate analysis,  $\beta$-coefficient | | Unknown | 6 |
| Llyod et al. 1998, USA[43] | n: 86;  age: 16.5-17.5;  female: 100.0% | Cross-sectional | Dietary record | Unknown | Girls | Fruits | n.s. | Multiple regression,  $\beta$-coefficient | | No | 4 |
| Michels et al. 2015, Multiple European countries[19] | n: 387;  age: 12.5-17.5;  female: n.a. | Cross-sectional | FFQ | No | no | Ready to eat cereals | n.s. ^a^ | Linear regression, Estimated marginal means | | Yes | 1 |
| Nobre et al. 2013, Brazil[20] | n: 227;  age: 4-5;  female: n.a. | Cross-sectional | FFQ | No | no | “Mixed dietary pattern“ (representing a typical Brazilian diet) | n.s. ^a^ | Multivariate poisson regression, Adjusted prevalence ratios | | Unknown | 1 |
| Ochoa-Avilés et al. 2014, Euqador[21] | n: 334;  age: 10-16;  female: n.a. | Cross-sectional | 24h recall |  | no | “Rice-rich non-animal fat pattern”  “Wheat-dense animal-fat pattern” | n.s.  n.s. | Linear regression,  $\beta$-% | |  | 2 |
| Rinaldi et al. 2012, Brazil[22] | n: 147;  age: 4-11 female: 51.7% | Cross-sectional | 24h recall | Yes | no | Dairy products  (full fat)  Carbohydrates  Proteins  SFA  Fat  MUFA  PUFA  Cholesterol  Fiber  Cereals  Meat  Legumes  Vegetables  Fruits  Sugar, sweet food  Oils and fats | n.s.  n.s.  n.s.  n.s.  n.s.  n.s.  n.s.  n.s.  n.s.  n.s.  n.s.  n.s.  n.s.  n.s.  n.s.  n.s. | Multiple linear regression,  $\beta$-coefficient | | Yes | 3 |
| Royo-Bordonada et al. 2003, Spain[23] | n: 1112;  age: 6-7;  female: 49.9% | Cross-sectional | FFQ | Unknown | no | “Dietary variety index” | n.s. | Partial correlation analysis,  Partial correlation coefficient | | No | 7 |
| Royo-Bordonada et al. 2006, Spain[24] | n:1112;  age: 6-7;  female: 49.9% | Cross-sectional | FFQ | Unknown | no | SFA | n.s. ^a^ | T-test,  Mean | | Yes | 5 |
| Sanchez-Bayle et al. 2008, Spain[25] | n: 673;  age: 6;  female: 47.7% | Cross-sectional | 24h recall | Unknown | no | Fat  SFA  MUFA  PUFA  Carbohydrates  Proteins | positive* ^a^  negative* ^a^  positive* ^a^  n.s. ^a^  n.s. ^a^  n.s. ^a^ | ANOVA,  Differences in means | | Unknown | 3 |
| Scaglioni et al. 2004, Italy[26] | n: 105;  age: 8;  female: 41.0% | Cross-sectional | FFQ; 24h recall | Unknown | no | High pasta, low red meat vs. Low pasta, high red meat | n.s. | Mann-Whitney U test, Mean | | Unknown | 2 |
| Shang et al. 2012, China[27] | n: 6974;  age: 6-13;  female: 49.0% | Cross-sectional | FFQ; 24h recall | No | no | SSB vs. milk, vs. other beverages | n.s.^a^ | General linear model, Mean differences | | Unknown | 6 |
| Song et al. 2015, Korea[28] | n: 2209;  age: 10-18;  female: 47.3% | Cross-sectional | 24h recall | Unknown | Boys  Girls | Carbohydrates  White rice  Carbohydrates  White rice | n.s. ^a^  n.s. ^a^  negative* ^a^  negative***^a^ | Multivariate linear regression,  Quartiles | | No | 8 |
| Steffen et al. 2003, USA[29] | n: 285;  age: 13, 15;  female: 45.6% | Prospective cohort study | FFQ | Unknown | no | Whole grains | n.s. ^a^ | Multiple linear regression, Adjusted mean values | | Yes | 4 |
| Takada et al. 1998, Japan[30] | n: 457;  age: 10;  female: 45.1% | Cross-sectional | FFQ | No | no | “Japanese diet score” | n.s. | Multiple linear regression,  $\beta$-coefficient | | Unknown | 0 |
| Truthmann et al. 2012, Germany[44] | n: 5198;  age: 12-17;  female: 49.1% | Cross-sectional | FFQ | Yes | Boys  Girls | HFD  HuSKY  IFI  F & V Index  HFD  HuSKY  IFI  F & V Index | n.s.  n.s.  n.s.  n.s.  n.s.  n.s.  n.s.  n.s. | Linear regression,  $\beta$-coefficient | | No | 10 |
| Van Rompay et al. 2015, USA[31] | n: 613;  age: 8-15;  female: n.a. | Cross-sectional | FFQ | No | no | SSB | n.s. ^a^ | Linear regression and ANCOVA,  Adjusted least square means | | Yes | 6 |
| Vyncke et al. 2013, Multiple European Countries  [32] | n: 552;  age: 12.5-17.5;  female: 52.0% | Cross-sectional | 24h recall | Yes | Boys  Girls | DQI-A  DQI-A | n.s.  n.s. | Multilevel regression models,  $\beta$-coefficient | | Yes | 2 |
| Wajid et al. 1995, Kashmir  [33] | n: 314;  age: 5-14;  female: 46.2% | Cross-sectional | Dietary history | Unknown | no | Fat | n.s. ^a^ | Pearson correlation, Mean | | Unknown | 1 |
| Wang et al. 2013, Canada[34] | n: 548;  age: 8-10;  female: n.a. | Cross-sectional | 24h recall | Yes | no | SSB | n.s. | Multivariate linear regression analysis,  $\beta$-coefficient | | Yes | 4 |
| Zhu et al. 2014, USA[36] | n: 5124;  age: 2-18;  female: 51.3% | Cross-sectional | FFQ | Unknown | n: 3272 | Yoghurt | n.s. ^a^ | Multivariate linear regression,  Least square means | | Unknown | 6 |
|  | **Outcome: LDL-cholesterol** | | | | | | | | | | |
| Ambrosini et al. 2010, Australia[1] | n: 1139;  age: 14;  female: 47.9% | Cross-sectional | FFQ | Unknown | Boys  Girls | “Healthy dietary pattern”  “Western dietary pattern”  “Healthy dietary pattern”  “Western dietary pattern” | n.s. ^a^  n.s. ^a^  n.s. ^a^  n.s. ^a^ | | ANOVA,  Mean | No | 9 |
| Ambrosini et al. 2013, Australia[2] | n: 1366;  age: 14-17;  female: 48.3% | Prospective cohort study | FFQ | Yes | Boys  (n: 587)  Girls  (n: 537) | SSB  SSB | n.s. ^a^  n.s. ^a^ | | Linear regression,  % of change | Unknown | 7 |
| Appannah et al. 2015, Australia[3] | n: 1163;  age: 14-17;  female: 48.0% | Prospective cohort study | FFQ | Yes | Boys  Girls | “Energy dense, high fat and low fiber dietary pattern”  “Energy dense, high fat and low fiber dietary pattern” | n.s.  n.s. | | GEE,  $\beta$-coefficient | Yes | 6 |
| Au et al. 2012, USA[4] | n:148;  age: 9-15;  female: 58.8% | Cross-sectional | FFQ | Yes | no | SFA  MUFA  PUFA  Carbohydrates | n.s.  n.s.  n.s.  n.s. | | Linear regression,  $\beta$-coefficient | Yes | 3 |
| Bel-Serrat et al. 2014a,  Multiple European Countries[5] | n: 454;  age: 12.5-17.5;  female: 56.0% | Cross-sectional | 24h recall | Yes | no | Proteins  Carbohydrates  Fat | n.s.  n.s.  n.s. | | Multi-level regression,  $\beta$-coefficient | Unknown | 4 |
| Bel-Serrat et al. 2014b,  Multiple European countries[6] | n: 454;  age: 12.5-17.5;  female: 56.0% | Cross- sectional | 24h recall | Yes | Boys  Girls | Alanine  Glycine  Isoleucine  Leucine  Valine  Phenylalanine  Tryptophan  Tyrosine  Arginine  Histidine  Lysine  Asparagine acids  Glutamic acid  Serine  Threonine  Cysteine  Methionine  Proline  Alanine  Glycine  Isoleucine  Leucine  Valine  Phenylalanine  Tryptophan  Tyrosine  Arginine  Histidine  Lysine  Asparagine acids  Glutamic acid  Serine  Threonine  Cysteine  Methionine  Proline | n.s.  n.s.  n.s.  n.s.  n.s.  n.s.  n.s.  n.s.  n.s.  n.s.  n.s.  n.s.  n.s.  n.s.  n.s.  n.s.  n.s.  n.s.  -0.14*  n.s.  n.s.  n.s.  n.s.  n.s.  n.s.  n.s.  -0.14*  n.s.  n.s.  n.s.  n.s.  n.s.  n.s.  n.s.  n.s.  n.s. | | Multilevel linear regression,  $\beta$-coefficient | Yes | 4 |
| Bremer et al. 2009, USA[7] | n: 6967;  age: 12-19;  female: 48.9% | Cross-sectional | 24h recall | Unknown | Boys  Girls | SSB  SSB | n.s.  n.s. | | Linear regression,  $\beta$-coefficient | No | 7 |
| Chan et al. 2014a, China[9] | n: 200;  age: 12-16;  female: 51.0% | Cross-sectional | FFQ | Unknown | no | SSB | n.s. ^a^ | | Multiple linear regression, Multivariate adjusted difference | Unknown | 1 |
| Chan et al. 2015, Australia  [11] | n: 2262;  age: 14, 17  female: 50.4% | Prospective cohort study | FFQ | Unknown | n: 1547 | DGI-CA | n.s. | | Linear regression,  $\beta$-coefficient | Unknown | 7 |
| Day et al. 2009, USA[12] | n: 489;  age: 8, 11, 14;  female: 51.3% | Cross-sectional | FFQ | Yes | Boys  Girls | Fat  Fat | n.s. ^a^  n.s. ^a^ | | ANCOVA,  Mean | Yes | 2 |
| Franko et al. 2010, USA[41] | n: 2371;  age: 9-19;  female: 100.0% | Prospective cohort study | Dietary record | Yes | Girls | Cereal consumption | negative* ^a^ | | Mixed regression models, Mean | Yes | 8 |
| Hur et al. 2012, USA[14] | n: 4928;  age: 12-19;  female: 49.8% | Cross-sectional | 24h recall | Unknown | Boys  Girls | Whole grains  Whole grains | n.s. ^a^  n.s. ^a^ | | Multiple linear regression, Adjusted mean values | No | 8 |
| Kell et al. 2014, USA[15] | n: 320;  age: 7-12;  female: 46.9% | Cross-sectional | 24h recall | Unknown | no | Added sugars  Dietary fat | n.s.  n.s. | | Linear regression,  $\beta$-coefficient | Unknown | 4 |
| Kosova et al. 2013, USA[16] | n: 4880;  age: 3-11;  female: 49.3% | Cross-sectional | 24h recall | Unknown | Age 3-5  (n: 467)  Age 6-8  (n: 558)  Age 9-11  (n: 574) | SSB  SSB  SSB | 1.64*  n.s.  n.s. | | Linear regression, Adjusted  $\beta$-coefficient | No | 9 |
| Kuzawa et al. 2003, Philippines  [17] | n: 608  age: 14-16;  female: 50.7% | Prospective cohort study | 24h recall | Unknown | Boys  Girls | Fat  Fat | 0.13*  n.s. | | Partial correlation coefficiens | Yes | 3 |
| Lin et al. 2014, Multiple European countries  [18] | n: 1804;  age: 12.5-17.5;  female: 52.6% | Cross-sectional | 24h recall | Yes | no | Dietary fiber  WSF  WIF | n.s.  0.03*  n.s. | | GLM multivariate analysis,  $\beta$-coefficient | Unknown | 6 |
| Michels et al. 2015, Multiple European countries[19] | n: 387;  age: 12.5-17.5;  female: n.a. | Cross-sectional | FFQ | No | no | Ready to eat cereals | n.s. ^a^ | | Linear regression, Estimated marginal means | Yes | 1 |
| Nobre et al. 2013, Brazil[20] | n:227;  age: 4-5;  female: n.a. | Cross-sectional | FFQ | No | no | “Mixed dietary pattern“ (representing a typical Brazilian diet) | negative** ^a^ | | Multivariate poisson regression, Adjusted prevalence ratios | Unknown | 1 |
| Ochoa-Avilés et al. 2014, Euqador[21] | n: 334;  age: 10-16;  female: n.a. | Cross-sectional | 24h recall |  | Rural  (n: n.a.)  Urban  (n: n.a.) | Rice-rich non-animal fat pattern  Wheat-dense animal-fat pattern  Wheat-dense animal-fat pattern | n.s.  8.4*  n.s. | | Linear regression,  $\beta$-% |  | 2 |
| Rinaldi et al. 2012, Brazil[22] | n: 147;  age: 4-11 female: 51.7% | Cross-sectional | 24h recall | Yes | no | Full fat dairy products  Carbohydrates  Proteins  SFA  Fat  MUFA  PUFA  Cholesterol intake  Fiber  Cereals  Meat  Legumes  Vegetables  Fruits  Sugar, sweet food  Oils and fats | n.s.  n.s.  n.s.  n.s.  n.s.  n.s.  n.s.  n.s.  n.s.  n.s.  n.s.  n.s.  n.s.  n.s.  n.s.  n.s. | | Multiple linear regression,  $\beta$-coefficient | Yes | 3 |
| Royo-Bordonada et al. 2003, Spain[23] | n: 1112;  age: 6-7;  female: 49.9% | Cross-sectional | FFQ | Unknown | no | Dietary variety index | n.s. | | Partial correlation analysis,  Partial correlation coefficient | No | 7 |
| Royo-Bordonada et al. 2006, Spain[24] | n: 1112;  age: 6-7;  female: 49.9% | Cross-sectional | FFQ | Unknown | no | SFA | positive* ^a^ | | T-test,  Mean | Yes | 5 |
| Sanchez-Bayle et al. 2008, Spain[25] | n: 673;  age: 6;  female: 47.7% | Cross-sectional | 24h recall | Unknown | no | Fat  SFA  MUFA  PUFA  Carbohydrates  Proteins | positive*** ^a^  positive*** ^a^  negative*** ^a^  n.s. ^a^  negative*** ^a^  n.s. ^a^ | | ANOVA,  Differences in means | Unknown | 3 |
| Scaglioni et al. 2004, Italy[26] | n: 105;  age: 8;  female: 41.0% | Cross-sectional | FFQ; 24h recall | Unknown | no | High pasta, low red meat vs. Low pasta, high red meat | n.s. ^a^ | | Mann-Whitney U test, Mean | Unknown | 2 |
| Shang et al. 2012, China[27] | n: 6974;  age: 6-13;  female: 49.0% | Cross-sectional | FFQ; 24h recall | No | no | SSB; vs. milk, vs. other beverages | n.s.^a^ | | General linear model, Mean differences | Unknown | 6 |
| Steffen et al. 2003, USA[29] | n: 285;  age: 13, 15;  female: 45.6% | Prospective cohort study | FFQ | Unknown | no | Whole grains | n.s. ^a^ | | Multiple linear regression, Adjusted mean values | Yes | 4 |
| Takada et al. 1998, Japan[30] | n: 457;  age: 10;  female: 45.1% | Cross-sectional | FFQ | No | no | Japanese diet score | -0.95* | | Multiple linear regression, $\beta$-coefficient | Unknown | 0 |
| Vyncke et al. 2013, Multiple European Countries[32] | n: 552;  age: 12.5-17.5;  female: 52.0% | Cross-sectional | 24h recall | Yes | Boys  Girls | DQI-A  DQI-A | n.s.  n.s. | | Multilevel regression models,  $\beta$-coefficient | Yes | 2 |
| Wajid et al. 1995, Kashmir  [33] | n: 314;  age: 5-14;  female: 46.2% | Cross-sectional | Dietary history | Unknown | no | Fat | n.s. ^a^ | | Pearson correlation, Mean | Unknown | 1 |
| Washi & Ageib 2010, Saudi Arabia[35] | n: 239;  age: 13-18;  female: 53.1% | Cross-sectional | FFQ | Unknown | no | Fat | 13.43* | | Chi-2-Test,  Chi-2-Value | Yes | 2 |
| Zhu et al. 2014, USA[36] | n: 5124;  age: 2-18;  female: 51.3% | Cross-sectional | FFQ | Unknown | n: 1265 | Yoghurt | n.s. ^a^ | | Multivariate linear regression,  Least square means | Unknown | 6 |
|  | **Outcome: Fasting insulin** | | | | | | | | | | |
| Ambrosini et al. 2010, Australia[1] | n: 1139;  age: 14;  female: 47.9% | Cross-sectional | FFQ | Unknown | Boys  Girls | “Healthy dietary pattern”  “Western dietary pattern”  “Healthy dietary pattern”  “Western dietary pattern” | n.s. ^a^  n.s. ^a^  n.s. ^a^  n.s. ^a^ | | ANOVA,  Mean | No | 9 |
| Ambrosini et al. 2013, Australia[2] | n: 1366;  age: 14-17;  female: 48.3% | Prospective cohort study | FFQ | Yes | Boys  (n: 564)  Girls  (n: 519) | SSB  SSB | n.s. ^a^  n.s. ^a^ | | Linear regression,  % of change | Unknown | 7 |
| Appannah et al. 2015, Australia[3] | n: 1163;  age: 14-17;  female: 48.0% | Prospective cohort study | FFQ | Yes | Boys  Girls | “Energy dense, high fat and low fiber dietary pattern”  “Energy dense, high fat and low fiber dietary pattern” | 3.0*  3.0* | | GEE,  $\beta$-coefficient (%) | Yes | 6 |
| Casazza et al. 2009a, USA[46] | n: 250;  age: 7-12;  female: 48.9% | Cross-sectional | 24h recall | Yes | no | Fat  Carbohydrates  Proteins  Sugar  fiber intake  SFA  MUFA  PUFA | n.s.  -0.10*  n.s.  n.s.  n.s.  n.s.  n.s.  n.s. | | Linear regression, Standardized parameter estimate | Unknown | 3 |
| Chan et al. 2015, Australia  [11] | n: 2262;  age: 14; 17  female: 50.4% | Prospective cohort study | FFQ | Unknown | n: 1479 | DGI-CA | -0.03* | | Linear regression,  $\beta$-coefficient | Unknown | 7 |
| Cook et al. 2014, USA[47] | n: 175;  age: 8-18;  female: 68.6% | Cross-sectional | 24h recall | Yes | no | Vegetables  No starchy vegetables  Nutrient rich vegetables | n.s. ^a^  n.s. ^a^  n.s. ^a^ | | ANOVA, Mean | Yes | 3 |
| Hur et al. 2012, USA[14] | n: 4928;  age:12-19;  female: 49.4% | Cross-sectional | 24h recall | Unknown | Boys  Girls | Whole grains  Whole grains | significant ^a^  negative* ^a^ | | Multiple linear regression, Adjusted mean values | No | 8 |
| Jimenez-Pavon et al. 2013, Multiple European countries[48] | n: 637;  age: 12.5-17.5;  female: 54.5% | Cross-sectional | 24h recall | Yes | Boys  Girls | DQI-A  DQI-A | n.s.  n.s. | | Multiple linear regression, Standardized  $\beta$-coefficient | Unknown | 2 |
| Lin et al. 2014, Multiple European countries[18] | n: 1804;  age: 12.5-17.5;  female: 52.6% | Cross-sectional | 24h recall | Yes | no | Dietary fiber  WSF  WIF | n.s.  n.s.  n.s. | | GLM multivariate analysis,  $\beta$-coefficient | Unknown | 6 |
| Michels et al. 2015, Multiple European countries[19] | n: 387;  age: 12.5-17.5;  female: n.a. | Cross-sectional | FFQ | No | no | Ready to eat cereals | n.s. ^a^ | | Linear regression, Estimated marginal means | Yes | 1 |
| Scaglioni et al. 2004, Italy[26] | n: 105;  age: 8;  female: 41.0% | Cross-sectional | FFQ;  24h recall | Unknown | no | High pasta, low red meat vs. Low pasta, high red meat | significant ^a^ | | Mann-Whitney U test, Mean | Unknown | 2 |
| Song et al. 2015, Korea[28] | n: 2209;  age: 10-18;  female: 47.3% | Cross-sectional | 24h recall | Unknown | Boys  Girls | Carbohydrates  White rice  Carbohydrates  White rice | n.s. ^a^  n.s. ^a^  n.s. ^a^  positive** ^a^ | | Linear regression, Quartiles | No | 8 |
| Steffen et al. 2003, USA[29] | n: 285;  age: 13, 15;  female: 45.6% | Prospective cohort study | FFQ | Unknown | no | Whole grains | n.s. ^a^ | | Multiple linear regression, Adjusted mean values | Yes | 4 |
| Wang et al. 2013, Canada[34] | n: 457;  age: 8-10;  female: n.a. | Prospective cohort study | 24h recall | Unknown | no | Added sugars (solid sources)  Added sugars (liquid sources) | n.s.  2.26** | | Linear regression,  $\beta$-coefficient | Yes | 4 |
| White et al. 2012, USA[49] | n: 774;  age: 16-17;  female: 100.0% | Prospective cohort study | Dietary record | Unknown | no | SFA  MUFA  PUFA  Carbohydrates  Sucrose  Starch  Soluble fiber  Insoluble fiber | n.s.  n.s.  -0.84*  n.s.  n.s.  n.s.  n.s.  n.s. | | Multiple linear regression,  $\beta$-coefficient | Yes | 5 |
| Zhu et al. 2014, USA[36] | n: 5124;  age: 2-18;  female: 51.3% | Cross-sectional | FFQ | Unknown | n: 913 | Yoghurt | negative*** ^a^ | | Linear regression,  Least square means | Unknown | 6 |
|  | **Outcome: Fasting glucose** | | | | | | | | | | |
| Ambrosini et al. 2010, Australia[1] | n: 1139;  age: 14;  female: 47.9% | Cross-sectional | FFQ | Unknown | Boys  Girls | “Healthy dietary pattern”  “Western dietary pattern”  “Healthy dietary pattern”  “Western dietary pattern” | negative* ^a^  n.s. ^a^  negative* ^a^  n.s. ^a^ | | ANOVA,  Mean | No | 9 |
| Ambrosini et al. 2013, Australia[2] | n: 1366;  age: 14-17;  female: 48.3% | Prospective cohort study | FFQ | Yes | Boys  (n: 587)  Girls  (n: 537) | SSB  SSB | n.s. ^a^  n.s. ^a^ | | Linear regression,  % of change | Unknown | 7 |
| Appannah et al. 2015, Australia[3] | n: 1163;  age: 14-17;  female: 48.0% | Prospective cohort study | FFQ | Yes | Boys  Girls | “Energy dense, high fat and low fiber dietary pattern”  “Energy dense, high fat and low fiber dietary pattern” | 0.04*  n.s. | | GEE,  $\beta$-coefficient | Yes | 6 |
| Casazza et al. 2009b, USA[8] | n: 202;  age: 7-12;  female: 47.0% | Cross-sectional | 24h recall | Unknown | no | Fat  Carbohydrates  Proteins | -0.36*  0.49*  -0.43* | | Linear regression, Standardized  $\beta$-coefficient | Yes | 3 |
| Chan et al. 2014b, China[10] | n: 2727;  age: 12-16;  female: 51.3% | Cross-sectional | FFQ | Unknown | Boys  Girls | SSB  SSB | n.s.  n.s. | | Linear regression,  $\beta$-coefficient | No | 6 |
| Chan et al. 2015, Australia  [11] | n: 2262;  age: 14, 17;  female: 50.4% | Prospective cohort study | FFQ | Unknown | n: 1478 | DGI-CA | n.s. | | Linear regression,  $\beta$-coefficient | Unknown | 7 |
| Cook et al. 2014, USA  [47] | n: 175;  age: 8-18;  female: 68.6% | Cross-sectional | 24h recall | Yes | no | Vegetables  No starchy vegetables  Nutrient rich vegetables | n.s. ^a^  n.s. ^a^  n.s. ^a^ | | ANOVA,  Mean | Yes | 3 |
| Donin et al. 2014, England[50] | n: 1841  age: 9-10;  female: n.a. | Cross-sectional | 24h recall | Yes | no | Fat  SFA  MUFA  PUFA  Carbohydrates  Sugars  Starch  No starch polysaccharides  Proteins | n.s.  n.s.  n.s.  n.s.  n.s.  n.s.  n.s.  n.s.  n.s. | | Multilevel linear regression,  % of change | Yes | 7 |
| Hong et al. 2009, South Korea[13] | n: 246;  age: 12-13;  female: 47.6% | Cross-sectional | Dietary record | Unknown | no | Carbohydrates  Proteins  Fat | n.s.  n.s.  n.s. | | Partial correlation analysis, Partial correlation coefficient | Unknown | 2 |
| Hur et al. 2012, USA[14] | n: 4928;  age:12-19;  female: 49.4% | Cross-sectional | 24h recall | Unknown | Boys  Girls | Whole grains  Whole grains | n.s. ^a^  n.s. ^a^ | | Multiple linear regression, Adjusted mean values | No | 8 |
| Lin et al. 2014, Multiple European countries[18] | n: 1804;  age: 12.5-17.5;  female: 52.6% | Cross-sectional | 24h recall | Yes | no | Dietary fiber  WSF  WIF | n.s.  -0.01*  n.s. | | GLM multivariate analysis,  $\beta$-coefficient | Unknown | 6 |
| Michels et al. 2015, Multiple European countries[19] | n: 387;  age: 12.5-17.5;  female: n.a. | Cross-sectional | FFQ | No | no | Ready to eat cereals | n.s. ^a^ | | Linear regression, Estimated marginal means | Yes | 1 |
| Ochoa-Avilés et al. 2014, Euqador  [21] | n: 334;  age: 10-16;  female: n.a. | Cross-sectional | 24h recall |  | Rural  (n.a.);  Urban  (n.a.) | “Rice-rich non-animal fat pattern”  “Rice-rich non-animal fat pattern”  “Wheat-dense animal-fat pattern” | n.s.  3.3**  n.s. | | Linear regression,  $\beta$-% |  | 2 |
| Royo-Bordonada et al. 2003, Spain[23] | n: 1112;  age: 6-7;  female: 49.9% | Cross-sectional | FFQ | Unknown | no | “Dietary variety index | n.s. | | Partial correlation analysis,  Partial correlation coefficient | No | 7 |
| Royo-Bordonada et al. 2006, Spain[24] | n: 1112;  age: 6-7;  female: 49.9% | Cross-sectional | FFQ | Unknown | no | SFA | n.s. ^a^ | | T-test,  Mean | Yes | 5 |
| Scaglioni et al. 2004, Italy[26] | n: 105;  age: 8;  female: 41.0% | Cross-sectional | FFQ;  24h recall | Unknown | no | High pasta, low red meat vs. Low pasta, high red meat | n.s. | | Mann-Whitney U test, Mean | Unknown | 2 |
| Shang et al. 2012, China[27] | n: 6974;  age: 6-13;  female: 49.0% | Cross-sectional | FFQ; 24h recall | No | no | SSB vs. milk, vs. other beverages | n.s.^a^ | | General linear model, Mean differences | Unknown | 6 |
| Song et al. 2015, Korea[28] | n: 2209;  age: 10-18;  female: 47.3% | Cross-sectional | 24h recall | Unknown | Boys  Girls | Carbohydrates  White rice  Carbohydrates  White rice | n.s. ^a^  n.s. ^a^  n.s. ^a^  n.s. ^a^ | | Linear regression, Quartiles | No | 8 |
| Steffen et al. 2003, USA[29] | n: 285;  age: 13, 15;  female: 45.6% | Prospective cohort study | FFQ | Unknown | no | Whole grains | n.s. ^a^ | | Multiple linear regression, Adjusted mean values | Yes | 4 |
| Wang et al. 2014, Canada[51] | n: 457;  age: 8-10;  female: n.a. | Prospective cohort study | 24h recall | Unknown | no | Added sugars (solid sources)  Added sugars (liquid sources) | n.s.  0.04** | | Linear regression,  $\beta$-coefficient | Yes | 4 |
| White et al. 2012, USA[49] | n: 774;  age: 16-17;  female: 100.0% | Prospective cohort study | Dietary record | Unknown | no | SFA  MUFA  PUFA  Carbohydrates  Sucrose  Starch  Soluble fiber  Insoluble fiber | n.s.  n.s.  n.s.  n.s.  n.s.  n.s.  n.s.  n.s. | | Multiple linear regression,  $\beta$-coefficient | Yes | 5 |
| Zhu et al. 2014, USA[36] | n: 5124;  age: 2-18;  female: 51.3% | Cross-sectional | FFQ | Unknown | no | Yoghurt | n.s. ^a^ | | Linear regression,  Least square means | Unknown | 6 |
|  | **Outcome: (HOMA-) insulin resistance** | | | | | | | | | | |
| Ambrosini et al. 2010, Australia[1] | n: 1139;  age: 14;  female: 48.0% | Cross-sectional | FFQ | Unknown | Boys  Girls | “Healthy dietary pattern”  “Western dietary pattern”  “Healthy dietary pattern”  “Western dietary pattern” | n.s. ^a^  n.s. ^a^  n.s. ^a^  n.s. ^a^ | | ANOVA,  Mean | No | 9 |
| Ambrosini et al. 2013, Australia[2] | n: 1366;  age: 14-17;  female: 48.3% | Prospective cohort study | FFQ | Yes | Boys  (n: 564)  Girls  (n: 519) | SSB  SSB | n.s. ^a^  n.s. ^a^ | | Linear regression,  % of change | Unknown | 7 |
| Appannah et al. 2015, Australia[3] | n: 1163;  age: 14-17;  female: 48.0% | Prospective cohort study | FFQ | Yes | Boys  Girls | “Energy dense, high fat and low fiber dietary pattern”  “Energy dense, high fat and low fiber dietary pattern” | 4.0*  4.0* | | GEE, $\beta$-coefficient(%) | Yes | 6 |
| Bremer et al. 2009, USA[7] | n: 6967;  age: 12-19;  female: 48.9% | Cross-sectional | 24h recall | Unknown | Boys  Girls | SSB  SSB | n.s.  0.07* | | Linear regression,  $\beta$-coefficient | No | 7 |
| Chan et al. 2015, Australia  [11] | n: 2262;  age: 14, 17;  female: 50.4% | Prospective cohort study | FFQ | Unknown | n: 1464 | DGI-CA | -0.004** | | Linear regression,  $\beta$-coefficient | Unknown | 7 |
| Cook et al. 2014, USA[47] | n: 175;  age: 8-18;  female: 68.6% | Cross-sectional | 24h recall | Yes | no | Vegetables  No starchy vegetables  Nutrient rich vegetables | n.s. ^a^  n.s. ^a^  n.s. ^a^ | | ANOVA, Mean | Yes | 3 |
| Donin et al. 2014, England[50] | n: 1841  age: 9-10;  female: n.a. | Cross-sectional | 24h recall | Yes | no | Fat  SFA  MUFA  PUFA  Carbohydrates  Sugars  Starch  No starch polysaccharides  Proteins | n.s.  n.s.  n.s.  n.s.  n.s.  n.s.  n.s.  n.s.  n.s. | | Multilevel linear regression,  % of change | Yes | 7 |
| Hirschler et al. 2008, Argentina[52] | n: 365;  age: 8-12;  female: 48.0% | Cross-sectional | FFQ | Unknown | no | Milk | -0.14* | | Linear regression,  $\beta$-coefficient | Unknown | 1 |
| Jimenez-Pavon et al. 2013, Multiple European countries[48] | n: 637;  age: 12.5-17.5;  female: 54.5% | Cross-sectional | 24h recall | Yes | Boys  Girls | DQI-A  DQI-A | n.s.  n.s. | | Multiple linear regression, Standardized  $\beta$-coefficient | Unknown | 2 |
| Jimenez-Pavon et al. 2014, Multiple European countries  [53] | n: 794;  age: 12.5-17.5;  female: 54.4% | Cross-sectional | 24h recall | Yes | Boys  Girls | DQI-A  DQI-A | n.s.  n.s. | | Logistic regression,  Odds ratio | Unknown | 2 |
| Karatzi et al. 2014, Greece[54] | n: 1912;  age: 9-13;  female: 51.4% | Cross-sectional | 24h recall | Unknown | no | Pattern 1: Fried potatoes, red meat, SSB;  Pattern 2: Processed meat, cheese;  Pattern 3: Margarine, sweets, savory snacks;  Pattern 4: Legumes, Fruits;  Pattern 5: Egg, fish | n.s.  n.s.  0.08***  n.s.  n.s. | | Multiple linear regression, Logistic regression, Standardized  $\beta$-coefficient | No | 8 |
| Kondaki et al. 2012, Multiple European countries  [55] | n: 546;  age: 12.5-17.5;  female: 54.6% | Cross-sectional | FFQ | Unknown | no | SSB  White bread  Brown bread | significant ^a^  n.s.  n.s. | | Multiple linear regression,  $\beta$-coefficient | Unknown | 4 |
| Kynde et al. 2009, Denmark[56] | n: 651;  age: 8-16;  female: 62.5% | Prospective study | 24 hour recall; food record | Unknown | 1. Boys  1.1 Children school  1.2 Adolescents  2. Girls  2.1 Children school  2.2 Adolescents | Total sugar  Added sugars  Non added sugars  Starch  Fiber  Total sugar  Added sugars  Non added sugars  Starch  Fiber  Total sugar  Added sugars  Non added sugars  Starch  Fiber  Total sugar  Added sugars  Non added sugars  Starch  Fiber | n.s.  n.s.  n.s.  n.s.  n.s.  n.s.  n.s.  n.s.  n.s.  n.s.  0.23*  n.s.  n.s.  n.s.  -1.28*  n.s.  n.s.  n.s.  n.s.  n.s. | | Linear regression,  $\beta$-coefficient | Unknown | 5 |
| Lopez Alarcon et al. 2014, Mexico[57] | n: 229;  age: 10-18;  female: 46.7% | Cross-sectional | 24h recall | Unknown | no | Proteins  Carbohydrates  Lipids  Fiber  SFA | n.s. ^a^  mixed ^a^  mixed ^a^  mixed ^a^  n.s. ^a^ | | Logistic regression,  Odds ratio | Yes | 2 |
| Michels et al. 2015, Multiple European countries  [19] | n: 387;  age: 12.5-17.5;  female: n.a. | Cross-sectional | FFQ | No | no | Ready to eat cereals | n.s. ^a^ | | Linear regression, Estimated marginal means | Yes | 1 |
| Romero-Polvo et al. 2012, Mexico[58] | n: 916;  age: 7-18;  female: 50.8% | Cohort study | FFQ |  | no | Western dietary pattern  Prudent dietary pattern  High protein/fat dietary pattern | positive^a^  n.s. ^a^  n.s. ^a^ | | Multiple logistic regressions,  Odd’s ratio |  | 5 |
| Sese et al. 2012, Multiple European Countries[59] | n: 826  age: 12.5-17.5;  female: 52.1% | Cross-sectional | FFQ | Yes | Boys  Girls | Fresh fruits  Vegetables  Dried fruits  Nuts, peanuts, seeds  Yoghurt, yoghurt products  Cheese products  Sweets, candy  Chocolate  Biscuits, cookies  Cake, muffins, pastries  Crisps, tortilla chips  Crackers, rice cakes, salty sticks  Popcorn  Meat based snacks  Bread, toast  Cereals  Oatmeal, porridge  Cereal bars  Sandwiches, toasties, pannini  Pizza  Hamburger  Hot dog, sausages  French fries  Pasta dishes  Pasta snack products  Milk  Chocolate milk  Soft drinks  Juices  Water  Fresh fruits  Vegetables  Dried fruits  Nuts, peanuts, seeds  Yoghurt, yoghurt products  Cheese products  Sweets, candy  Chocolate  Biscuits, cookies  Cake, muffins, pastries  Crisps, tortilla chips  Crackers, rice cakes, salty sticks  Popcorn  Meat based snacks  Bread, toast  Bowl of cereal  Oatmeal, porridge  Cereal bars  Sandwiches, toasties, pannini  Pizza  Hamburger  Hot dog, sausages  French fries  Pasta dishes  Pasta snack products  Milk  Chocolate milk  Soft drinks  Juices  Water | n.s.^a^  n.s.^a^  n.s.^a^  n.s.^a^  n.s. ^a^  n.s.^a^  n.s.^a^  n.s.^a^  n.s.^a^  n.s.^a^  n.s.^a^  n.s.^a^  n.s.^a^  n.s.^a^  n.s.^a^  n.s.^a^  n.s.^a^  n.s.^a^  n.s.^a^  significant**^a^**  significant**^a^**  n.s.^a^  n.s.^a^  n.s.^a^  n.s.^a^  n.s.^a^  n.s.^a^  n.s.^a^  n.s.^a^  n.s.^a^  n.s.^a^  n.s.^a^  n.s.^a^  significant**^a^**  n.s.^a^  n.s.^a^  n.s.^a^  significant**^a^**  n.s.^a^  n.s.^a^  n.s.^a^  n.s.^a^  n.s.^a^  significant**^a^**  n.s.^a^  n.s.^a^  n.s.^a^  n.s.^a^  n.s.^a^  n.s.^a^  significant**^a^**  n.s.^a^  n.s.^a^  n.s.^a^  n.s.^a^  n.s.^a^  n.s.^a^  significant**^a^**  significant**^a^**  n.s.^a^ | | One way ANOVA,  Mean | Unknown | 1 |
| Song et al. 2015, Korea[28] | n: 2209;  age: 10-18;  female: 47.3% | Cross-sectional | 24h recall | Unknown | Boys  Girls | Carbohydrates  White rice  Carbohydrates  White rice | n.s. ^a^  n.s. ^a^  n.s. ^a^  positive** ^a^ | | Linear regression, Quartiles | No | 8 |
| Wang et al. 2013, Canada[34] | n: 548;  age: 8-10;  female: n.a. | Cross-sectional | 24h recall | Yes | no | SSB | n.s. | | Linear regression analysis,  $\beta$-coefficient | Yes | 4 |
| Wang et al. 2014, Canada[51] | n: 457;  age: 8-10;  female: n.a. | Prospective cohort study | 24h recall | Unknown | no | Added sugars (solid sources)  Added sugars (liquid sources) | n.s.  0.09** | | Linear regression,  $\beta$-coefficient | Yes | 4 |
| White et al. 2012, USA[49] | n: 774;  age: 16-17;  female: 100.0% | Prospective cohort study | Dietary record | Unknown | no | SFA  MUFA  PUFA  Carbohydrates  Sucrose  Starch  Soluble fiber  Insoluble fiber | n.s.  n.s.  n.s.  n.s.  n.s.  n.s.  n.s.  n.s. | | Multiple linear regression,  $\beta$-coefficient | Yes | 5 |
| Zhu et al. 2014, USA[36] | n: 5124;  age: 2-18;  female: 51.3% | Cross-sectional | FFQ | Unknown | n: 3769 | Yoghurt | negative*** ^a^ | | Linear regression,  Least square means | Unknown | 6 |
|  | **Outcome: insulin sensitivity** | | | | | | | | | | |
| Casazza et al. 2009a, USA[46] | n: 250;  age: 7-12;  female: 48.9% | Cross-sectional | 24h recall | Yes | no | Fat  Carbohydrates  Proteins  Sugar  Fiber  SFA  MUFA  PUFA | n.s.  n.s.  n.s.  n.s.  n.s.  n.s.  n.s.  n.s. | | Linear regression, Standardized parameter estimate | Unknown | 3 |
| Cook et al. 2014, USA[47] | n: 175;  age: 8-18;  female: 68.6% | Cross-sectional | 24h recall | Yes | no | Vegetables  No starchy vegetables  Nutrient rich vegetables | n.s. ^a^  n.s. ^a^  positive* ^a^ | | ANOVA, Mean | Yes | 3 |
| Davis et al. 2007, USA[60] | n: 120;  age: 10-17;  female: 43.3% | Cross-sectional | 24h recall | Unknown | no | Sugar | -0.02* | | Hierarchical multiple regression analyses, Unstandar-dized  $\beta$-coefficient | Yes | 3 |
| Forbes et al. 2013, Canada[61] | n: 378;  age: 10-14;  female: 60.1% | Cross- sectional; Cohort study | 24h recall | Yes | Boys  Girls | Sugar  Fiber  Fruits/Vegetables  Fat  Sugar  Fiber  Fruits/Vegetables  Fat | n.s.  n.s.  n.s.  n.s.  n.s.  -0.18*  n.s.  n.s. | | Least squares multiple regression analysis,  $\beta$-coefficient | Yes | 3 |
| Steffen et al. 2003, USA[29] | n: 285;  age: 13, 15;  female: 45.6% | Prospective cohort study | FFQ | Unknown | no | Whole grains | positive* ^a^ | | Multiple linear regression, Adjusted mean values | Yes | 4 |
|  | **Outcome: C-reactive Protein** | | | | | | | | | | |
| Aeberli et al. 2006, Switzerland  [62] | n: 79;  age: 6-14;  female: 46.8% | Cross-sectional | 24h recall; 1d food record | Yes | no | Fat  Fat (% energy)  SFA  PUFA  MUFA  Dairy products  Meat  Plant oils  Animal fat | 0.28**  0.28**  0.24*  0.21*  0.27*  n.s.  n.s.  0.24*  n.s. | Multiple regression and ANCOVA,  $\beta$-coefficient | | Yes | 3 |
| Au et al. 2012, USA[4] | n: 148;  age: 9-15;  female: 58.8% | Cross-sectional | FFQ | Yes | no | SFA  MUFA  PUFA  Carbohydrates | n.s.  n.s.  n.s.  n.s. | Linear regression,  $\beta$-coefficient | | Yes | 3 |
| Chan et al. 2015, Australia  [11] | n: 2262;  age: 14, 17;  female: 50.4% | Prospective cohort study | FFQ | Unknown | n: 1458 | DGI-CA | n.s. | Linear regression,  $\beta$-coefficient | | Unknown | 7 |
| Gonzalez Gil et al. 2015, Multiple European countries  [63] | n: 3884;  age: 6-9;  female: 50.9% | Cross-sectional | FFQ | No | Boys  Gils | Raw vegetables  Raw vegetables | 0.7*  n.s. | Multilevel ordinal logistic regression,  Odds ratio | | Unknown | 5 |
| Holt et al. 2009, USA[64] | n: 285;  age: 13-17;  female: 45.6% | Cross –sectional; Cohort study | FFQ | Yes | no | Fruits (no fruit juice)  Fruit juice  Vegetables  French fries  Legumes  Fruits/Vegetables | -0.19**  n.s.  n.s.  n.s.  n.s.  -0.15* | Spearman partial correlation coefficients, Correlation coefficient | | Yes | 4 |
| Hur et al. 2012, USA[14] | n: 4928;  age: 12-19;  female: 49.4% | Cross-sectional | 24h recall | Unknown | Boys  Girls | Whole grains  Whole grains | n.s. ^a^  significant ^a^ | Multiple linear regression, Adjusted mean values | | No | 8 |
| Kosova et al. 2013, USA[16] | n: 4880;  age: 3-11;  female: 49.3% | Cross-sectional | 24h recall | Unknown | Age 3-5  (n: 1227)  Age 6-8  (n: 1316)  Age 9-11  (n: 1375) | SSB  SSB  SSB | n.s.  n.s.  0.01* | Linear regression, Adjusted  $\beta$-coefficient | | No | 9 |
| Lin et al. 2014, Multiple European countries  [18] | n: 1804;  age: 12.5-17.5;  female: 52.6% | Cross-sectional | 24h recall | Yes | no | Dietary fiber  Soluble Fiber  Insoluble Fiber | n.s.  n.s.  n.s. | GLM multivariate analysis,  $\beta$-coefficient | | Unknown | 6 |
| Qureshi et al. 2009, USA[65] | n: 4110;  age: 5-16;  female: 49.9% | Cross-sectional | 24h recall | Unknown | no | Dairy products  Milk  Cheese  Yoghurt  Grains  Refined grains  Whole grains  Fruits  Citrus, melon, berries  Other fruits  Vegetables  Non starchy vegetables  Dark green vegetables  Deep yellow/orange vegetables  Tomatoes  Starchy vegetables  Legumes  Potatoes  Meat/other Proteins  Red meat  White meat  Other Proteins sources | significant ^a^  significant ^a^  n.s. ^a^  n.s. ^a^  significant ^a^  significant ^a^  n.s. ^a^  n.s. ^a^  significant ^a^  n.s. ^a^  significant ^a^  significant ^a^  n.s. ^a^  n.s. ^a^  significant ^a^  significant ^a^  n.s. ^a^  n.s. ^a^  n.s. ^a^  n.s. ^a^  n.s. ^a^  n.s. ^a^ | ANOVA, Mean differences | | No | 7 |
| Thomas et al. 2008, UK[66] | n: 164;  age: 12-13;  female: 54.3% | Cross-sectional | FFQ , food diary | Yes | Boys  Girls | Fat  SFA  Fat  SFA | n.s.  n.s.  n.s.  n.s. | Pearson correlation, Partial correlation coefficient | | No | 4 |
| Truthmann et al. 2012, Germany[44] | n: 5198;  age:12-17;  female: 49.1% | Cross-sectional | FFQ | Yes | Boys  (n: 2554)  Girls  (n: 2438) | HFD  HuSKY  IFI  F & V Index  HFD  HuSKY  IFI  F & V Index | n.s.  -8.20*  n.s.  n.s.  n.s.  n.s.  -14.00**  n.s. | Linear regression,  $\beta$-coefficient | | No | 10 |
| Vyncke et al. 2013, Multiple European Countries[32] | n: 552;  age: 12.5-17.5;  female: 52.0% | Cross-sectional | 24h recall | Yes | Boys  Girls | DQI-A  DQI-A | n.s.  n.s. | Multilevel regression models,  $\beta$-coefficient | | Yes | 2 |
| Zhu et al. 2014, USA[36] | n: 5124;  age: 2-18;  female: 51.3% | Cross-sectional | FFQ | Unknown | n: 3769 | Yoghurt | n.s. ^a^ | Linear regression,  Least square means | | Unknown | 6 |
|  | **Outcome: systolic blood pressure** | | | | | | | | | | |
| Ambrosini et al. 2013, Australia[2] | n: 1366;  age: 14-17;  female: 48.3% | Prospective cohort study | FFQ | Yes | Boys  Girls | SSB  SSB | n.s. ^a^  n.s. ^a^ | | Linear regression,  % of change | Unknown | 7 |
| Aounallah-Skhiri et al. 2011, Tunisia[67] | n: 1019;  age: 15-19;  female: 57.6% | Cross-sectional | FFQ | Yes | Boys  Girls | “Modern dietary pattern”  “Meat & fish dietary pattern”  “Modern dietary pattern”  “Meat & fish dietary pattern” | n.s. ^a^  n.s. ^a^  n.s. ^a^  n.s. ^a^ | | Linear/ Logistic regression,  Mean difference | Yes | 8 |
| Bobridge et al. 2013, Australia  [68] | n: 814;  age: 13.0-14.9;  female: 48.5% | Cross-sectional | Dietary record | Yes | Boys  Girls | Fructose  Fructose | n.s.  n.s. | | Linear regression, Standardized  $\beta$-coefficient | Yes | 4 |
| Bremer et al. 2009, USA[7] | n: 6967;  age: 12-19;  female: 48.9% | Cross-sectional | 24h recall | Unknown | Boys  Girls | SSB  SSB | n.s.  0.38* | | Linear regression,  $\beta$-coefficient | No | 7 |
| Boreham et al. 1999, Northern Ireland[45] | n: 454;  age: 12-15;  female: 50.7% | Longitudinal cohort study | Dietary history | Unknown | Boys | Carbohydrates | 0.27* | | GEE,  $\beta$-coefficient | No | 4 |
| Casazza et al. 2009b, USA[8] | n: 202;  age: 7-12;  female: 47.0% | Cross-sectional | 24h recall | Unknown | no | Fat  Carbohydrates  Proteins | 0.05*  n.s.  -0.15* | | Linear regression, Standardized  $\beta$-coefficient | Yes | 3 |
| Chan et al. 2014b, China[10] | n: 2727;  age: 12-16;  female: 51.3% | Cross-sectional | FFQ | Unknown | Boys  Girls | SSB  SSB | 1.6*  n.s. | | Linear regression,  $\beta$-coefficient | No | 6 |
| Chan et al. 2015, Australia  [11] | n: 2262;  age: 14, 17;  female: 50.4% | Prospective cohort study | FFQ | Unknown | no | DGI-CA | n.s. | | Linear regression,  $\beta$-coefficient | Unknown | 7 |
| Coelho et al. 2015, Brazil[69] | n: 738;  age: 6-14;  female: 51.3% | Cross-sectional | FFQ |  | age: 6-9  age: 10-14 | RFS adapted by Coelho et al. 2012  RFS adapted by Coelho et al. 2012 | -0.11*  n.s. | | Linear regression,  $\beta$-coefficient |  | 2 |
| Colin Ramirez et al. 2009, Mexico[70] | n: 1239;  age: 8-10 years;  female: 49.5% | Cross-sectional | 24h recall | No | no | Fat  SFA  MUFA | n.s.  n.s.  n.s. | | Logistic regression,  $\beta$-coefficient | Yes | 5 |
| Day et al. 2009, USA[12] | n: 489;  age: 8, 11, 14;  female: 51.3% | Cross-sectional | FFQ | Yes | Boys  Girls | Fat  Fat | n.s. ^a^  significant**^a^** | | ANCOVA,  Mean | Yes | 2 |
| De Moraes et al. 2015, Multiple European countries[71] | n: 1605;  age: 12.5-17.5;  female: 51.9% | Cross-sectional | 24h recall |  | Boys  Girls | Vegetal Proteins  Animal Proteins  Total Proteins  Alanine  Glycine  Isoleucine  Leucine  Valine  Phenilalanine  Tryptophane  Tyrosine  Arginine  Histidine  Lysine  Asparaginic acid  Glutamic acid  Serine  Theorine  Cysteine  Methionine  Proline  Vegetal Proteins  Animal Proteins  Total Proteins  Alanine  Glycine  Isoleucine  Leucine  Valine  Phenilalanine  Tryptophane  Tyrosine  Arginine  Histidine  Lysine  Asparaginic acid  Glutamic acid  Serine  Theorine  Cysteine  Methionine  Proline | n.s.  n.s.  n.s.  n.s.  n.s.  n.s.  n.s.  n.s.  n.s.  n.s.  n.s.  n.s.  1.01**  n.s.  n.s.  n.s.  n.s.  n.s.  n.s.  n.s.  n.s.  n.s.  n.s.  n.s.  n.s.  n.s.  n.s.  n.s.  n.s.  n.s.  4.41**  -2.14*  n.s.  1.13**  n.s.  n.s.  n.s.  n.s.  n.s.  n.s.  n.s.  n.s. | | Multiple linear regression,  $\beta$-coefficient |  | 6 |
| Gopinath et al. 2014, Australia  [72] | n: 888;  age: baseline 12, follow up 17;  female: 49.0% | Cohort study | FFQ | Unknown | Boys  Girls | Total dairy  Milk  Cheese  Yoghurt  Total dairy  Milk  Cheese  Yoghurt | n.s.  n.s.  n.s.  n.s.  n.s.  n.s.  -7.18**  n.s. | | Linear regression,  $\beta$-coefficient | Yes | 5 |
| Hojhabrimanesh et al. 2015, Iran[73] | n: 557;  age: 12-14.9;  female: 56.4% | Cross-sectional | FFQ | Yes | no | Mixed dietary pattern score  Western Dietary pattern score  Prudent Dietary pattern Score | n.s. ^a^  significant^a^  n.s. ^a^ | | ANCOVA,  Mean | No | 8 |
| Hong et al. 2009, South Korea[13] | n: 246;  age: 12-13;  female: 47.6% | Cross-sectional | Dietary record | Unknown | no | Carbohydrates  Proteins  Fat | n.s.  n.s.  n.s. | | Partial correlation analysis, Partial correlation coefficient | Unknown | 2 |
| Hur et al. 2012, USA[14] | n: 4928;  age: 12-19;  female: 49.4% | Cross-sectional | 24h recall | Unknown | Boys  Girls | Whole grains  Whole grains | n.s. ^a^  n.s. ^a^ | | Multiple linear regression, Adjusted mean values | No | 8 |
| Kelishadi et al. 2006, Iran[74] | n: 21111;  age: 6-18;  female: 51.4% | Cross-sectional | FFQ | Yes | no | Dairy products  Sweets/Candy  Solid hydrogenated fat  Fast food | -0.43**  0.32**  1.64**  1.38** | | Linear regression,  $\beta$-coefficient  Logistic regression,  Odds ratio | No | 8 |
| Kell et al. 2014, USA[15] | n: 320;  age: 7-12;  female: 46.9% | Cross-sectional | 24h recall | Unknown | no | Added sugars | n.s. | | Linear regression,  $\beta$-coefficient | Unknown | 4 |
| Kollias et al. 2009, Greece[75] | n: 558;  age: 12-17;  female: 50.0% | Cross-sectional | FFQ | No | Boys  Girls | Meat  Legumes  Fruits/Vegetables  Milk  Snacks  Meat  Legumes  Fruits/Vegetables  Milk  Snacks | n.s.  n.s.  n.s.  n.s.  n.s.  n.s.  n.s.  n.s.  n.s.  n.s. | | Multiple regression analysis,  $\beta$-coefficient | Yes | 1 |
| Lazarou et al. 2009, Republic of Cyprus[76] | n: 622;  age: 9-13;  female: 50.8% | Cross-sectional | FFQ | Unknown | no | Food E-KINDEX Score | 0.43* | | Logistic regression,  Odds ratio | Unknown | 6 |
| Moore et al. 2005, USA[77] | n: 91;  age:  Dietary intake assessment at age 3.0-5.9 years and 6.0-11.9 years, blood pressure assessment at age 10.0-12.9 years;  female: n.a. | Prospective study | Dietary record | Yes | no | Fruits/Vegetables  Dairy intake | n.s. ^a^  n.s. ^a^ | | ANOCVA,  Mean | Unknown | 3 |
| O Sullivan et al. 2012, Australia  [78] | n: 814;  age: 13-15;  female: 48.5% | Cohort study | Dietary record | Unknown | Boys  Girls | Fat  PUFA  Total omega 3 FA  Alpha linoleic acid  Long chain omega 3 FA  EPA  DPA  DHA  Total omega 6 FA  Linoleic acid  Arachidonic acid  Fat  PUFA  Total omega 3 FA  Alpha linoleic acid  Long chain omega 3 FA  EPA  DPA  DHA  Total omega 6 FA  Linoleic acid  Arachidonic acid | -0.12*  -0.15*  -0.12*  n.s.  n.s.  -0.11*  n.s.  n.s.  -0.11*  -0.11*  n.s.  n.s.  n.s.  n.s.  n.s.  n.s.  n.s.  n.s.  n.s.  n.s.  n.s.  n.s. | | Linear regression, Standardized  $\beta$-coefficient | Yes | 4 |
| Ochoa-Avilés et al. 2014, Euqador[21] | n: 334;  age: 10-16;  female: n.a. | Cross-sectional | 24h recall |  | Rural  (n: n.a.)  Urban  (n: n.a.) | “Rice-rich non-animal fat pattern”  “Wheat-dense animal-fat pattern”  “Wheat-dense animal-fat pattern” | n.s.  n.s.  n.s. | | Linear regression,  $\beta$-% |  | 2 |
| Shang et al. 2012, China[27] | n: 6974;  age: 6-13;  female: 49.0% | Cross-sectional | FFQ, 24h recall | No | no | SSB vs. milk, vs. other beverages | n.s. ^a^ | | General linear model, Mean differences | Unknown | 6 |
| Shi et al. 2014, Germany[79] | n: 435;  age: 4-18;  female: 51.3% | Prospective cohort study | Dietary record | Yes | no | Fruits/Vegetables  intake | n.s. | | Linear mixed effects regression models,  $\beta$-coefficient | Yes | 5 |
| Song et al. 2015, Korea[28] | n: 2209;  age: 10-18;  female: 47.3% | Cross-sectional | 24h recall | Unknown | Boys  Girls | Carbohydrates  White rice intake  Carbohydrates  White rice | n.s. ^a^  n.s. ^a^  n.s. ^a^  n.s. ^a^ | | Linear regression, Quartiles | No | 8 |
| Souza et al. 2016, Brazil[80] | n: 488  age: 9-16;  female: 49.4% | Cross-sectional | FFQ | Yes | no | Soft drinks | significant ^a^ | | Linear regression,  Estimated mean | Unknown | 2 |
| Steffen et al. 2003, USA[29] | n: 285;  age: 13, 15;  female: 45.6% | Prospective cohort study | FFQ | Unknown | no | Whole grains | n.s. ^a^ | | Multiple linear regression, Adjusted mean values | Yes | 4 |
| Sugiyama et al. 2007, USA[81] | n: 4508;  age: 12-19;  female: 49.1% | Cross-sectional | 24h recall | Unknown | no | Carbohydrates  Proteins  SFA  MUFA  PUFA  Fiber | n.s.  n.s.  n.s.  n.s.  n.s.  n.s. | | Multiple linear regression,  $\beta$-coefficient | No | 7 |
| Truthmann et al. 2012, Germany[44] | n: 5198;  age: 12-17;  female: 49.1% | Cross-sectional | FFQ | Yes | Boys  Girls | HFD  HuSKY  IFI  F & V Index  HFD  HuSKY  IFI  F & V Index | n.s.  n.s.  n.s.  n.s.  n.s.  n.s.  n.s.  n.s. | | Linear regression,  $\beta$-coefficient | No | 10 |
| Wang et al. 2013, Canada[34] | n: 548;  age: 8-10;  female: n.a. | Cross-sectional | 24h recall | Yes | no | SSB | 0.58** | | Linear regression,  $\beta$-coefficient | Yes | 4 |
| Zhu et al. 2014, USA[36] | n: 5124;  age: 2-18;  female: 51.3% | Cross-sectional | FFQ | Unknown | n: 2868 | Yoghurt | n.s. ^a^ | | Linear regression,  Least square means | Unknown | 6 |
|  | **Outcome: diastolic blood pressure** | | | | | | | | | | |
| Ambrosini et al. 2013, Australia[2] | n: 1366;  age: 14-17;  female: 48.3% | Prospective cohort study | FFQ | Yes | Boys  Girls | SSB  SSB | n.s. ^a^  n.s. ^a^ | | Linear regression,  % of change | Unknown | 7 |
| Aounallah-Skhiri et al. 2011, Tunisia[67] | n: 1019;  age: 15-19;  female: 57.6% | Cross-sectional | FFQ | Yes | Boys  Girls | “Modern dietary pattern”  “Meat & fish dietary pattern”  “Modern dietary pattern”  “Meat & fish dietary pattern” | n.s. ^a^  n.s. ^a^  n.s. ^a^  n.s. ^a^ | | Linear/ Logistic regression,  Mean difference | Yes | 8 |
| Bobridge et al. 2013, Australia  [68] | n: 814;  age: 13.0-14.9;  female: 48.5% | Cross-sectional | Dietary record | Yes | Boys  Girls | Fructose  Fructose | n.s.  n.s. | | Yes |  | 4 |
| Bremer et al. 2009, USA[7] | n: 6967;  age: 12-19;  female: 48.9% | Cross-sectional | 24h recall | Unknown | Boys  Girls | SSB  SSB | n.s.  n.s. | | Linear regression,  $\beta$-coefficient | No | 7 |
| Chan et al. 2014b, China[10] | n: 2727;  age: 12-16;  female: 51.3% | Cross-sectional | FFQ | Unknown | Boys  Girls | SSB  SSB | n.s.  n.s. | | Linear regression,  $\beta$-coefficient | No | 6 |
| Chan et al. 2015, Australia  [11] | n: 2262;  age: 14, 17;  female: 50.4% | Prospective cohort study | FFQ | Unknown | n:1701 | DGI-CA | n.s. | | Linear regression,  $\beta$-coefficient | Unknown | 7 |
| Colin Ramirez et al. 2009, Mexico[70] | n: 1239;  age: 8-10 years;  female: 49.5% | Cross-sectional | 24h recall | No | no | Fat  SFA  MUFA | 2.61**  n.s.  n.s. | | Logistic regression,  $\beta$-coefficient | Yes | 5 |
| Day et al. 2009, USA[12] | n:489;  age: 8, 11, 14;  female: 51.3% | Cross-sectional | FFQ | Yes | Boys  Girls | Fat  Fat | n.s. ^a^  n.s. ^a^ | | ANCOVA,  Mean | Yes | 2 |
| De Moraes et al. 2015, Multiple European countries[71] | n: 1605;  age: 12.5-17.5;  female: 51.9% | Cross-sectional | 24h recall |  | Boys  Girls | Plant Proteins  Animal Proteins  Total Proteins  Alanine  Glycine  Isoleucine  Leucine  Valine  Phenilalanine  Tryptophane  Tyrosine  Arginine  Histidine  Lysine  Asparaginic acid  Glutamic acid  Serine  Theorine  Cysteine  Methionine  Proline  Plant Proteins  Animal Proteins  Total Proteins  Alanine  Glycine  Isoleucine  Leucine  Valine  Phenilalanine  Tryptophane  Tyrosine  Arginine  Histidine  Lysine  Asparaginic acid  Glutamic acid  Serine  Theorine  Cysteine  Methionine  Proline | -1.16*  -1.82*  n.s.  n.s.  n.s.  n.s.  n.s.  n.s.  n.s.  n.s.  n.s.  n.s.  n.s.  n.s.  n.s.  n.s.  n.s.  n.s.  n.s.  n.s.  n.s.  n.s.  n.s.  n.s.  -1.41*  n.s.  n.s.  n.s.  n.s.  n.s.  n.s.  n.s.  n.s.  n.s.  n.s.  n.s.  n.s.  n.s.  n.s.  n.s.  2.78***  n.s. | | Linear regression,  $\beta$-coefficient |  | 6 |
| Gopinath et al. 2014, Australia  [72] | n: 888;  age: baseline 12, follow up 17;  female: 49.0% | Cohort study | FFQ | Unknown | Boys  Girls | Total dairy  Milk  Cheese  Yoghurt  Total dairy  Milk  Cheese  Yoghurt | n.s.  n.s.  n.s.  n.s.  -1.04*  -1.14*  -5.28**  n.s. | | Linear regression,  $\beta$-coefficient | Yes | 5 |
| Hojhabrimanesh et al. 2015, Iran[73] | n: 557;  age: 12-14.9;  female: 56.4% | Cross-sectional | FFQ | Yes | no | “Mixed dietary pattern score”  “Western Dietary pattern score”  “Prudent Dietary pattern Score” | n.s. ^a^  n.s. ^a^  n.s. ^a^ | | ANCOVA,  Mean | No | 8 |
| Hong et al. 2009, South Korea[13] | n: 246;  age: 12-13;  female: 47.6% | Cross-sectional | Dietary record | Unknown | no | Carbohydrates  Proteins  Fat | n.s.  n.s.  n.s. | | Partial correlation analysis, Partial correlation coefficient | Unknown | 2 |
| Kelishadi et al. 2006, Iran[74] | n: 21111;  age: 6-18;  female: 51.4% | Cross-sectional | FFQ | Yes | no | Whole grain bread  Vegetables  Sweets/Candy  Trans-fat  Fast food | -0.47**  -0.44**  0.41*  1.61**  1.41** | | Linear regression,  $\beta$-coefficient;  Logistic regression,  Odds ratio | No | 8 |
| Kell et al. 2014, USA[15] | n: 320;  age: 7-12;  female: 46.9% | Cross-sectional | 24h recall | Unknown | no | Added sugars | 0.02* | | Linear regression,  $\beta$-coefficient | Unknown | 4 |
| Kollias et al. 2009, Greece[75] | n: 558;  age: 12-17;  female: 50.0% | Cross-sectional | FFQ | No | Boys  Girls | Meat  Legumes  Fruits/Vegetables  Milk  Snacks  Meat  Legumes  Fruits/Vegetables  Milk  Snacks | n.s.  n.s.  n.s.  -2.14*  n.s.  n.s.  n.s.  n.s.  n.s.  n.s. | | Multiple regression analysis,  $\beta$-coefficient | Yes | 1 |
| Lazarou et al. 2009, Republic of Cyprus[76] | n: 622;  age: 9-13;  female: 50.8% | Cross-sectional | FFQ |  | no | Food E-KINDEX Score | 0.52* | | Logistic regression,  Odds ratio |  | 6 |
| Moore et al. 2005, USA[77] | n: 91;  age at intake assessment: 3.0-5.9 ; 6.0-11.9, age at outcome assessment: 10.0-12.9  female: n.a. | Prospective study | Dietary record | Yes | no | Fruits/Vegetables  Dairy | n.s. ^a^  n.s. ^a^ | | ANOCVA,  Mean | Unknown | 3 |
| O Sullivan et al. 2012, Australia  [78] | n: 814;  age: 13-15;  female: 48.5% | Cohort study | Dietary record | Unknown | Boys  Girls | Fat  PUFA  Total n3-FA  Alpha linoleic acid  LC-n3-FA  EPA  DPA  DHA  Total n6-FA  Linoleic acid  Arachidonic acid  Fat  PUFA  Total n3-FA  Alpha linoleic acid  LC-n3-FA  EPA  DPA  DHA  Total n6-FA  Linoleic acid  Arachidonic acid | n.s.  n.s.  n.s.  n.s.  -0.15*  -0.15*  n.s.  -0.14*  n.s.  n.s.  -0.12*  n.s.  n.s.  n.s.  n.s.  n.s.  n.s.  n.s.  n.s.  n.s.  n.s.  n.s. | | Linear regression, Standardized  $\beta$-coefficient | Yes | 4 |
| Ochoa-Avilés et al. 2014, Euqador  [21] | n: 334;  age: 10-16;  female: n.a. | Cross-sectional | 24h recall |  | no | “Rice-rich non-animal fat pattern”  “Wheat-dense animal-fat pattern” | n.s.  n.s. | | Linear regression,  $\beta$-% |  | 2 |
| Shang et al. 2012, China[27] | n: 6974;  age: 6-13;  female: 49.0% | Cross-sectional | FFQ; 24h recall | No | no | SSB vs. milk, vs. other beverages | n.s. ^a^ | | General linear model, Mean differences | Unknown | 6 |
| Shi et al. 2014, Germany  [79] | n: 435;  age: 4-18;  female: 51.3% | Prospective cohort study | Dietary record | Yes | no | Fruits/Vegetables | n.s. | | Linear mixed effects regression models,  $\beta$-coefficient | Yes | 5 |
| Song et al. 2015, Korea[28] | n: 2209;  age: 10-18;  female: 47.3% | Cross-sectional | 24h recall | Unknown | Boys  Girls | Carbohydrates  White rice  Carbohydrates  White rice | n.s. ^a^  n.s. ^a^  n.s. ^a^  n.s. ^a^ | | Linear regression, Quartiles | No | 8 |
| Souza et al. 2016, Brazil[80] | n: 488  age: 9-16;  female: 49.4% | Cross-sectional | FFQ | Yes | no | Soft drinks | significant ^a^ | | Linear regression,  Estimated mean | Unknown | 2 |
| Sugiyama et al. 2007, USA[81] | n: 4508;  age: 12-19;  female: 49.1% | Cross-sectional | 24h recall | Unknown | no | Carbohydrates  Proteins  SFA  MUFA  PUFA  Fiber | n.s.  n.s.  n.s.  0.09*  n.s.  n.s. | | Multiple linear regression,  $\beta$-coefficient | No | 7 |
| Truthmann et al. 2012, Germany[44] | n: 5198;  age: 12-17;  female: 49.1% | Cross-sectional | FFQ | Yes | Boys  Girls | HFD  HuSKY  IFI  F&V Index  HFD  HuSKY  IFI  F&V Index | n.s.  n.s.  n.s.  n.s.  n.s.  n.s.  0.33*  0.26* | | Linear regression,  $\beta$-coefficient | No | 10 |
| Zhu et al. 2014, USA[36] | n: 5124;  age: 2-18;  female: 51.3% | Cross-sectional | FFQ | Unknown | no | Yoghurt | n.s. ^a^ | | Linear regression,  Least square means | Unknown | 6 |
|  | **Outcome: Leptin** | | | | | | | | | | |
| Aeberli et al. 2006, Switzerland  [62] | n: 79;  age: 6-14;  female: 46.8% | Cross-sectional | 24h recall; 1d food record | Yes | no | Fat  Fat (% energy)  SFA  PUFA  MUFA  Dairy products  Meat  Plant oils  Animal fat | n.s.  n.s.  n.s.  n.s.  n.s.  n.s.  0.17*  n.s.  n.s. | | ANCOVA,  $\beta$-coefficient | Yes | 3 |
| Lin et al. 2014, Multiple European countries[18] | n: 1804;  age: 12.5-17.5;  female: 52.6% | Cross-sectional | 24h recall | Yes | no | Fiber  Soluble fiber  Insoluble fiber | n.s.  n.s.  n.s. | | GLM multivariate analysis,  $\beta$-coefficient | Unknown | 6 |
|  | **Outcome: HbA1c** | | | | | | | | | | |
| Donin et al. 2014, England[50] | n: 1841  age: 9-10;  female: n.a. | Cross-sectional | 24h recall | Yes | no | Fat  SFA  MUFA  PUFA  Carbohydrates  Sugars  Starch  No starch polysaccharides  Proteins | n.s.  n.s.  n.s.  n.s.  n.s.  n.s.  n.s.  n.s.  n.s. | | Multilevel linear regression,  % of change | Yes | 7 |
| Truthmann et al. 2012, Germany[44] | n: 5198;  age: 12-17;  female: 49.1% | Cross-sectional | FFQ | Yes | Boys  (n: 2646)  Girls  (n:2552) | HFD  HuSKY  IFI  F&V Index  HFD  HuSKY  IFI  F&V Index | n.s.  n.s.  -0.01*  n.s.  n.s.  n.s.  n.s.  n.s. | | Linear regression,  $\beta$-coefficient | No | 10 |

Effect estimates: p<: 0.001: ***; p<: 0.01: **; p<: 0.05: *

Quality Score: 0-4 : low; 5-8 : moderate; 9-11 : high

n.s.: not significant; n.a. : not available

a) Categorized intake variable, please see original manuscript for further details

**Abbreviations:**

ANCOVA: Analysis of Covariance

DGI-CA: Dietary Guideline Index for Children and Adolescents

DHA: Decosahexaenoic acid

DPA: Decosapentaenoic acid

DQI-A: Dietary quality index

EPA: Eicosapentaenoic acid

FA: Fatty acids

FFQ: Food Frequency Questionnaire

F&V Index: Fruit and Vegetable Index

GEE: Generalised estimation equasions

HFD: Healthy Food Diversity Index

HuSKY: Healthy Nutrition Score for Kids and Youth

IFI: Indicator Food Index

MUFA: Monounsaturated Fatty Acids

PUFA: Polyunsaturated Fatty Acids

RFS: Recommended Food Score

SFA: Saturated Fatty Acids

SSB: Sugar sweetened beverages

**References**

1. Ambrosini GL, Huang RC, Mori TA, Hands BP, O'Sullivan TA, de Klerk NH, Beilin LJ, Oddy WH: Dietary patterns and markers for the metabolic syndrome in Australian adolescents. Nutrition Metabolism and Cardiovascular Diseases 2010, 20:274-283.

2. Ambrosini GL, Oddy WH, Huang RC, Mori TA, Beilin LJ, Jebb SA: Prospective associations between sugar-sweetened beverage intakes and cardiometabolic risk factors in adolescents. Am J Clin Nutr 2013, 98:327-334.

3. Appannah G, Pot GK, Huang RC, Oddy WH, Beilin LJ, Mori TA, Jebb SA, Ambrosini GL: Identification of a dietary pattern associated with greater cardiometabolic risk in adolescence. Nutr Metab Cardiovasc Dis 2015, 25:643-650.

4. Au LE, Economos CD, Goodman E, Houser RF, Must A, Chomitz VR, Morgan EH, Sacheck JM: Dietary intake and cardiometabolic risk in ethnically diverse urban schoolchildren. J Acad Nutr Diet 2012, 112:1815-1821.

5. Bel-Serrat S, Mouratidou T, Huybrechts I, Labayen I, Cuenca-Garcia M, Palacios G, Breidenassel C, Molnar D, Roccaldo R, Widhalm K, et al: Associations between macronutrient intake and serum lipid profile depend on body fat in European adolescents: the Healthy Lifestyle in Europe by Nutrition in Adolescence (HELENA) study. British Journal of Nutrition 2014, 112:2049-2059.

6. Bel-Serrat S, Mouratidou T, Huybrechts I, Cuenca-Garcia M, Manios Y, Gomez-Martinez S, Molnar D, Kafatos A, Gottrand F, Widhalm K, et al: The role of dietary fat on the association between dietary amino acids and serum lipid profile in European adolescents participating in the HELENA Study. Eur J Clin Nutr 2014, 68:464-473.

7. Bremer AA, Auinger P, Byrd RS: Relationship between insulin resistance-associated metabolic parameters and anthropometric measurements with sugar-sweetened beverage intake and physical activity levels in US adolescents: findings from the 1999-2004 National Health and Nutrition Examination Survey. Arch Pediatr Adolesc Med 2009, 163:328-335.

8. Casazza K, Dulin-Keita A, Gower BA, Fernandez JR: Differential influence of diet and physical activity on components of metabolic syndrome in a multiethnic sample of children. J Am Diet Assoc 2009, 109:236-244.

9. Chan TF, Lin WT, Chen YL, Huang HL, Yang WZ, Lee CY, Chen MH, Wang TN, Huang MC, Chiu YW, et al: Elevated serum triglyceride and retinol-binding protein 4 levels associated with fructose-sweetened beverages in adolescents. PLoS One 2014, 9:e82004.

10. Chan TF, Lin WT, Huang HL, Lee CY, Wu PW, Chiu YW, Huang CC, Tsai S, Lin CL, Lee CH: Consumption of sugar-sweetened beverages is associated with components of the metabolic syndrome in adolescents. Nutrients 2014, 6:2088-2103.

11. Chan She Ping-Delfos WL, Beilin LJ, Oddy WH, Burrows S, Mori TA: Use of the Dietary Guideline Index to assess cardiometabolic risk in adolescents. Br J Nutr 2015, 113:1741-1752.

12. Day RS, Fulton JE, Dai S, Mihalopoulos NL, Barradas DT: Nutrient intake, physical activity, and CVD risk factors in children: Project HeartBeat! Am J Prev Med 2009, 37:S25-33.

13. Hong HR, Kim SU, Kang HS: Physical activity and metabolic syndrome in Korean children. Int J Sports Med 2009, 30:677-683.

14. Hur IY, Reicks M: Relationship between whole-grain intake, chronic disease risk indicators, and weight status among adolescents in the National Health and Nutrition Examination Survey, 1999-2004. J Acad Nutr Diet 2012, 112:46-55.

15. Kell KP, Cardel MI, Bohan Brown MM, Fernandez JR: Added sugars in the diet are positively associated with diastolic blood pressure and triglycerides in children. Am J Clin Nutr 2014, 100:46-52.

16. Kosova EC, Auinger P, Bremer AA: The relationships between sugar-sweetened beverage intake and cardiometabolic markers in young children. J Acad Nutr Diet 2013, 113:219-227.

17. Kuzawa CW, Adair LS, Avila JL, Cadungog JH, Le NA: Atherogenic lipid profiles in Filipino adolescents with low body mass index and low dietary fat intake. Am J Hum Biol 2003, 15:688-696.

18. Lin Y, Huybrechts I, Vereecken C, Mouratidou T, Valtuena J, Kersting M, Gonzalez-Gross M, Bolca S, Warnberg J, Cuenca-Garcia M, et al: Dietary fiber intake and its association with indicators of adiposity and serum biomarkers in European adolescents: the HELENA study. Eur J Nutr 2014.

19. Michels N, De Henauw S, Breidenassel C, Censi L, Cuenca-Garcia M, Gonzalez-Gross M, Gottrand F, Hallstrom L, Kafatos A, Kersting M, et al: European adolescent ready-to-eat-cereal (RTEC) consumers have a healthier dietary intake and body composition compared with non-RTEC consumers. Eur J Nutr 2015, 54:653-664.

20. Nobre LN, Lamounier JA, Franceschini Sdo C: Sociodemographic, anthropometric and dietary determinants of dyslipidemia in preschoolers. J Pediatr (Rio J) 2013, 89:462-469.

21. Ochoa-Aviles A, Verstraeten R, Lachat C, Andrade S, Van Camp J, Donoso S, Kolsteren P: Dietary intake practices associated with cardiovascular risk in urban and rural Ecuadorian adolescents: a cross-sectional study. Bmc Public Health 2014, 14.

22. Rinaldi AE, de Oliveira EP, Moreto F, Gabriel GF, Corrente JE, Burini RC: Dietary intake and blood lipid profile in overweight and obese schoolchildren. BMC Res Notes 2012, 5:598.

23. Royo-Bordonada MA, Gorgojo L, Ortega H, Martin-Moreno JM, Lasuncion MA, Garces C, Gil A, Rodriguez-Artalejo F, de Oya M: Greater dietary variety is associated with better biochemical nutritional status in Spanish children: the Four Provinces Study. Nutr Metab Cardiovasc Dis 2003, 13:357-364.

24. Royo-Bordonada MA, Garces C, Gorgojo L, Martin-Moreno JM, Lasuncion MA, Rodriguez-Artalejo F, Fernandez O, de Oya M: Saturated fat in the diet of Spanish children: relationship with anthropometric, alimentary, nutritional and lipid profiles. Public Health Nutr 2006, 9:429-435.

25. Sanchez-Bayle M, Gonzalez-Requejo A, Pelaez MJ, Morales MT, Asensio-Anton J, Anton-Pacheco E: A cross-sectional study of dietary habits and lipid profiles. The Rivas-Vaciamadrid study. Eur J Pediatr 2008, 167:149-154.

26. Scaglioni S, Veduci E, Agostoni C, Vergani B, Stival G, Riva E, Giovannini M: Dietary habits and plasma fatty acids levels in a population of Italian children: is there any relationship? Prostaglandins Leukot Essent Fatty Acids 2004, 71:91-95.

27. Shang XW, Liu AL, Zhang Q, Hu XQ, Du SM, Ma J, Xu GF, Li Y, Guo HW, Du L, et al: Report on childhood obesity in China (9): sugar-sweetened beverages consumption and obesity. Biomed Environ Sci 2012, 25:125-132.

28. Song S, Paik HY, Song WO, Song Y: Metabolic syndrome risk factors are associated with white rice intake in Korean adolescent girls and boys. British Journal of Nutrition 2015, 113:479-487.

29. Steffen LM, Jacobs DR, Jr., Murtaugh MA, Moran A, Steinberger J, Hong CP, Sinaiko AR: Whole grain intake is associated with lower body mass and greater insulin sensitivity among adolescents. Am J Epidemiol 2003, 158:243-250.

30. Takada H, Harrell J, Deng S, Bandgiwala S, Washino K, Iwata H: Eating habits, activity, lipids and body mass index in Japanese children: the Shiratori Children Study. Int J Obes Relat Metab Disord 1998, 22:470-476.

31. Van Rompay MI, McKeown NM, Goodman E, Eliasziw M, Chomitz VR, Gordon CM, Economos CD, Sacheck JM: Sugar-Sweetened Beverage Intake Is Positively Associated with Baseline Triglyceride Concentrations, and Changes in Intake Are Inversely Associated with Changes in HDL Cholesterol over 12 Months in a Multi-Ethnic Sample of Children. J Nutr 2015, 145:2389-2395.

32. Vyncke KE, Huybrechts I, Dallongeville J, Mouratidou T, Van Winckel MA, Cuenca-Garcia M, Ottevaere C, Gonzalez-Gross M, Moreno LA, Kafatos AG, et al: Intake and serum profile of fatty acids are weakly correlated with global dietary quality in European adolescents. Nutrition 2013, 29:411-419 e411-413.

33. Wajid Ali S, Buch NA, Masood H: Serum lipid profile in Kashmiri children. Indian J Physiol Pharmacol 1995, 39:55-58.

34. Wang JW, Mark S, Henderson M, O'Loughlin J, Tremblay A, Wortman J, Paradis G, Gray-Donald K: Adiposity and glucose intolerance exacerbate components of metabolic syndrome in children consuming sugar-sweetened beverages: QUALITY cohort study. Pediatr Obes 2013, 8:284-293.

35. Washi SA, Ageib MB: Poor diet quality and food habits are related to impaired nutritional status in 13- to 18-year-old adolescents in Jeddah. Nutr Res 2010, 30:527-534.

36. Zhu Y, Wang H, Hollis JH, Jacques PF: The associations between yogurt consumption, diet quality, and metabolic profiles in children in the USA. Eur J Nutr 2014.

37. Akerblom HK, Viikari J, Uhari M, Rasanen L, Suoninen P, Pietikainen M, Pesonen E, Lahde PL, Dahl M, Dahlstrom S, et al.: A study of cardiovascular risk factors and their determinants in finnish children. Ann Clin Res 1984, 16:23-33.

38. Altwaijri YA, Day RS, Harrist RB, Dwyer JT, Ausman LM, Labarthe DR: Sexual maturation affects diet-blood total cholesterol association in children: Project HeartBeat! Am J Prev Med 2009, 37:S65-70.

39. Beck CC, Lopes AD, de Farias JC: Factors associated with serum lipids of adolescents from the Brazilian South. Revista De Nutricao-Brazilian Journal of Nutrition 2014, 27:35-43.

40. Boulton TJ, Magarey AM, Cockington RA: Serum lipids and apolipoproteins from 1 to 15 years: changes with age and puberty, and relationships with diet, parental cholesterol and family history of ischaemic heart disease. Acta Paediatr 1995, 84:1113-1118.

41. Franko DL, Albertson AM, Thompson DR, Barton BA: Cereal consumption and indicators of cardiovascular risk in adolescent girls. Public Health Nutrition 2011, 14:584-590.

42. Fukushima T, Hojo N, Isobe A, Gao T, Shiwaku K, Yamane Y: Food intake, serum lipids and amino acids of school children in agricultural communities in Japan. Eur J Clin Nutr 1999, 53:207-210.

43. Lloyd T, Chinchilli VM, Rollings N, Kieselhorst K, Tregea DF, Henderson NA, Sinoway LI: Fruit consumption, fitness, and cardiovascular health in female adolescents: the Penn State Young Women's Health Study. Am J Clin Nutr 1998, 67:624-630.

44. Truthmann J, Richter A, Thiele S, Drescher L, Roosen J, Mensink GB: Associations of dietary indices with biomarkers of dietary exposure and cardiovascular status among adolescents in Germany. Nutr Metab (Lond) 2012, 9:92.

45. Boreham C, Twisk J, van Mechelen W, Savage M, Strain J, Cran G: Relationships between the development of biological risk factors for coronary heart disease and lifestyle parameters during adolescence: The Northern Ireland Young Hearts Project. Public Health 1999, 113:7-12.

46. Casazza K, Dulin-Keita A, Gower BA, Fernandez JR: Relationships between reported macronutrient intake and insulin dynamics in a multi-ethnic cohort of early pubertal children. Int J Pediatr Obes 2009, 4:249-256.

47. Cook LT, O'Reilly GA, Goran MI, Weigensberg MJ, Spruijt-Metz D, Davis JN: Vegetable Consumption Is Linked to Decreased Visceral and Liver Fat and Improved Insulin Resistance in Overweight Latino Youth. J Acad Nutr Diet 2014.

48. Jimenez-Pavon D, Sese MA, Huybrechts I, Cuenca-Garcia M, Palacios G, Ruiz JR, Breidenassel C, Leclercq C, Beghin L, Plada M, et al: Dietary and lifestyle quality indices with/without physical activity and markers of insulin resistance in European adolescents: the HELENA study. Br J Nutr 2013, 110:1919-1925.

49. White J, Jago R, Thompson JL: Dietary risk factors for the development of insulin resistance in adolescent girls: a 3-year prospective study. Public Health Nutr 2014, 17:361-368.

50. Donin AS, Nightingale CM, Owen CG, Rudnicka AR, Jebb SA, Ambrosini GL, Stephen AM, Cook DG, Whincup PH: Dietary Energy Intake Is Associated With Type 2 Diabetes Risk Markers in Children. Diabetes Care 2014, 37:116-123.

51. Wang J, Light K, Henderson M, O'Loughlin J, Mathieu ME, Paradis G, Gray-Donald K: Consumption of added sugars from liquid but not solid sources predicts impaired glucose homeostasis and insulin resistance among youth at risk of obesity. J Nutr 2014, 144:81-86.

52. Hirschler V, Oestreicher K, Beccaria M, Hidalgo M, Maccallini G: Inverse association between insulin resistance and frequency of milk consumption in low-income Argentinean school children. J Pediatr 2009, 154:101-105.

53. Jimenez-Pavon D, Sese MA, Valtuena J, Cuenca-Garcia M, Gonzalez-Gross M, Gottrand F, Kafatos A, Manios Y, Widhalm K, de Henauw S, et al: Leptin, vitamin D, and cardiorespiratory fitness as risk factors for insulin resistance in European adolescents: gender differences in the HELENA Study. Appl Physiol Nutr Metab 2014, 39:530-537.

54. Karatzi K, Moschonis G, Barouti AA, Lionis C, Chrousos GP, Manios Y: Dietary patterns and breakfast consumption in relation to insulin resistance in children. The Healthy Growth Study. Public Health Nutr 2014:1-8.

55. Kondaki K, Grammatikaki E, Jimenez-Pavon D, De Henauw S, Gonzalez-Gross M, Sjostrom M, Gottrand F, Molnar D, Moreno LA, Kafatos A, et al: Daily sugar-sweetened beverage consumption and insulin resistance in European adolescents: the HELENA (Healthy Lifestyle in Europe by Nutrition in Adolescence) Study. Public Health Nutr 2013, 16:479-486.

56. Kynde I, Johnsen NF, Wedderkopp N, Bygbjerg IB, Helge JW, Heitmann BL: Intake of total dietary sugar and fibre is associated with insulin resistance among Danish 8-10- and 14-16-year-old girls but not boys. European Youth Heart Studies I and II. Public Health Nutr 2010, 13:1669-1674.

57. Lopez-Alarcon M, Perichart-Perera O, Flores-Huerta S, Inda-Icaza P, Rodriguez-Cruz M, Armenta-Alvarez A, Bram-Falcon MT, Mayorga-Ochoa M: Excessive refined carbohydrates and scarce micronutrients intakes increase inflammatory mediators and insulin resistance in prepubertal and pubertal obese children independently of obesity. Mediators Inflamm 2014, 2014:849031.

58. Romero-Polvo A, Denova-Gutierrez E, Rivera-Paredez B, Castanon S, Gallegos-Carrillo K, Halley-Castillo E, Borges G, Flores M, Salmeron J: Association between dietary patterns and insulin resistance in Mexican children and adolescents. Ann Nutr Metab 2012, 61:142-150.

59. Sese MA, Jimenez-Pavon D, Gilbert CC, Gonzalez-Gross M, Gottrand F, de Henauw S, Breidenassel C, Warnberg J, Widhalm K, Molnar D, et al: Eating behaviour, insulin resistance and cluster of metabolic risk factors in European adolescents. The HELENA study. Appetite 2012, 59:140-147.

60. Davis JN, Alexander KE, Ventura EE, Kelly LA, Lane CJ, Byrd-Williams CE, Toledo-Corral CM, Roberts CK, Spruijt-Metz D, Weigensberg MJ, Goran MI: Associations of dietary sugar and glycemic index with adiposity and insulin dynamics in overweight Latino youth. Am J Clin Nutr 2007, 86:1331-1338.

61. Forbes LE, Downs SM, Fraser SN, Majumdar SR, Ball GD, Plotnikoff RC, Wozny PD, Torrance BD, McCargar LJ, Lewanczuk RZ, McGavock JM: Anthropometric and dietary predictors of insulin sensitivity in 10- to 14-year-old boys and girls. Appl Physiol Nutr Metab 2013, 38:320-325.

62. Aeberli I, Molinari L, Spinas G, Lehmann R, l'Allemand D, Zimmermann MB: Dietary intakes of fat and antioxidant vitamins are predictors of subclinical inflammation in overweight Swiss children. Am J Clin Nutr 2006, 84:748-755.

63. Gonzalez-Gil EM, Santabarbara J, Russo P, Ahrens W, Claessens M, Lissner L, Bornhorst C, Krogh V, Iacoviello L, Molnar D, et al: Food intake and inflammation in European children: the IDEFICS study. Eur J Nutr 2015.

64. Holt EM, Steffen LM, Moran A, Basu S, Steinberger J, Ross JA, Hong CP, Sinaiko AR: Fruit and Vegetable Consumption and Its Relation to Markers of Inflammation and Oxidative Stress in Adolescents. Journal of the American Dietetic Association 2009, 109:414-421.

65. Qureshi MM, Singer MR, Moore LL: A cross-sectional study of food group intake and C-reactive protein among children. Nutr Metab (Lond) 2009, 6:40.

66. Thomas NE, Baker JS, Graham MR, Cooper SM, Davies B: C-reactive protein in schoolchildren and its relation to adiposity, physical activity, aerobic fitness and habitual diet. British Journal of Sports Medicine 2008, 42.

67. Aounallah-Skhiri H, Traissac P, El Ati J, Eymard-Duvernay S, Landais E, Achour N, Delpeuch F, Ben Romdhane H, Maire B: Nutrition transition among adolescents of a south-Mediterranean country: dietary patterns, association with socio-economic factors, overweight and blood pressure. A cross-sectional study in Tunisia. Nutr J 2011, 10:38.

68. Bobridge KS, Haines GL, Mori TA, Beilin LJ, Oddy WH, Sherriff J, O'Sullivan TA: Dietary fructose in relation to blood pressure and serum uric acid in adolescent boys and girls. J Hum Hypertens 2013, 27:217-224.

69. Coelho LG, Candido APC, Machado-Coelho GLL, de Freitas SN: Food habits and risk of cardiovascular disease in schoolchildren from Ouro Preto, Minas Gerais. Revista De Nutricao-Brazilian Journal of Nutrition 2015, 28:133-142.

70. Colin-Ramirez E, Castillo-Martinez L, Orea-Tejeda A, Villa Romero AR, Vergara Castaneda A, Asensio Lafuente E: Waist circumference and fat intake are associated with high blood pressure in Mexican children aged 8 to 10 years. J Am Diet Assoc 2009, 109:996-1003.

71. de Moraes ACF, Bel-Serrat S, Manios Y, Molnar D, Kafatos A, Cuenca-Garcia M, Huybrechts I, Sette S, Widhalm K, Stehle P, et al: Dietary protein and amino acids intake and its relationship with blood pressure in adolescents: the HELENA STUDY. European Journal of Public Health 2015, 25:450-456.

72. Gopinath B, Flood VM, Burlutsky G, Louie JC, Baur LA, Mitchell P: Dairy food consumption, blood pressure and retinal microcirculation in adolescents. Nutr Metab Cardiovasc Dis 2014, 24:1221-1227.

73. Hojhabrimanesh A, Akhlaghi M, Rahmani E, Amanat S, Atefi M, Najafi M, Hashemzadeh M, Salehi S, Faghih S: A Western dietary pattern is associated with higher blood pressure in Iranian adolescents. Eur J Nutr 2015.

74. Kelishadi R, Ardalan G, Gheiratmand R, Majdzadeh R, Delavari A, Heshmat R, Gouya MM, Razaghi EM, Motaghian M, Mokhtari MR, et al: Blood pressure and its influencing factors in a national representative sample of Iranian children and adolescents: the CASPIAN Study. Eur J Cardiovasc Prev Rehabil 2006, 13:956-963.

75. Kollias A, Antonodimitrakis P, Grammatikos E, Chatziantonakis N, Grammatikos EE, Stergiou GS: Trends in high blood pressure prevalence in Greek adolescents. J Hum Hypertens 2009, 23:385-390.

76. Lazarou C, Panagiotakos DB, Matalas AL: Foods E-KINDEX: A Dietary Index Associated with Reduced Blood Pressure Levels among Young Children: The CYKIDS Study. Journal of the American Dietetic Association 2009, 109:1070-1075.

77. Moore LL, Singer MR, Bradlee ML, Djousse L, Proctor MH, Cupples LA, Ellison RC: Intake of fruits, vegetables, and dairy products in early childhood and subsequent blood pressure change. Epidemiology 2005, 16:4-11.

78. O'Sullivan TA, Bremner AP, Beilin LJ, Ambrosini GL, Mori TA, Huang RC, Oddy WH: Polyunsaturated fatty acid intake and blood pressure in adolescents. J Hum Hypertens 2012, 26:178-187.

79. Shi L, Krupp D, Remer T: Salt, fruit and vegetable consumption and blood pressure development: a longitudinal investigation in healthy children. Br J Nutr 2014, 111:662-671.

80. Souza Bda S, Cunha DB, Pereira RA, Sichieri R: Soft drink consumption, mainly diet ones, is associated with increased blood pressure in adolescents. J Hypertens 2016, 34:221-225.

81. Sugiyama T, Xie D, Graham-Maar RC, Inoue K, Kobayashi Y, Stettler N: Dietary and lifestyle factors associated with blood pressure among U.S. adolescents. J Adolesc Health 2007, 40:166-172.
